# Magnetic Field-Driven Strategies for Biofilm Disruption: From Iron Oxide Nanoparticles to Adaptive Swarms of Magnetic Microrobots

**DOI:** 10.1021/acsnano.5c14390

**Published:** 2026-01-01

**Authors:** Maja Caf, Parvaneh Esmaeilnejad-Ahranjani, Jelena Kolosnjaj-Tabi, Jerica Sabotič, Aleš Berlec, Nika Zaveršek, Stane Pajk, Abida Zahirović, Muriel Golzio, Irena Milosevic, Slavko Kralj

**Affiliations:** 1 Department for Materials Synthesis, 61790 Jožef Stefan Institute, Ljubljana 1000, Slovenia; 2 Faculty of Pharmacy, 63721 University of Ljubljana, Ljubljana 1000, Slovenia; 3 54920 Institut de Pharmacologie et de Biologie Structurale (IPBS), Université de Toulouse, CNRS, Université Toulouse III-Paul Sabatier (UPS), Toulouse 31400, France; 4 Department of Biotechnology, 61790 Jožef Stefan Institute, Ljubljana 1000, Slovenia; 5 HEPIA, University of Applied Sciences of Western Switzerland (HES-SO), Geneva 1202, Switzerland

**Keywords:** antibiotic resistance, biofilm eradication, magnetic nanoparticles, magneto-mechanical actuation, magnetic microrobot swarms, microrobots, nanorobots, microrobotic superstructures, SPIONs

## Abstract

Biofilms, structured communities of microbial cells embedded in extracellular polymeric substances, are notorious for their resilience against conventional antimicrobial treatments. They contribute significantly to chronic infections and industrial biofouling, necessitating innovative strategies for their eradication. Magnetic iron oxide nanoparticles have emerged as a promising tool in combating biofilms due to their biocompatibility and unique physicochemical properties, which enable magnetic delivery of antibacterial agents, magnetic hyperthermia, magneto-mechanical actuation including mechanical biofilm disruption, and reversible dynamic magnetic assembly into hierarchical structures. This review describes developing stages of magnetic nanoscale weapons against biofilms ranging from individual iron oxide nanoparticles to complex hierarchical nanoparticle assemblies in the form of magnetic robots and their swarms. A vast array of possible antibiofilm and antibacterial functionalities originating from iron ions, individual iron oxide nanoparticles, spherical nanoparticle assemblies, magnetic robots, and swarms of robots are presented. Magnetic nanotools offer significant improvements and advantages over conventional methods for biofilm eradication, yet their successful future applications depend on addressing and overcoming critical material, biological, and engineering challenges.

## Graphical abstract

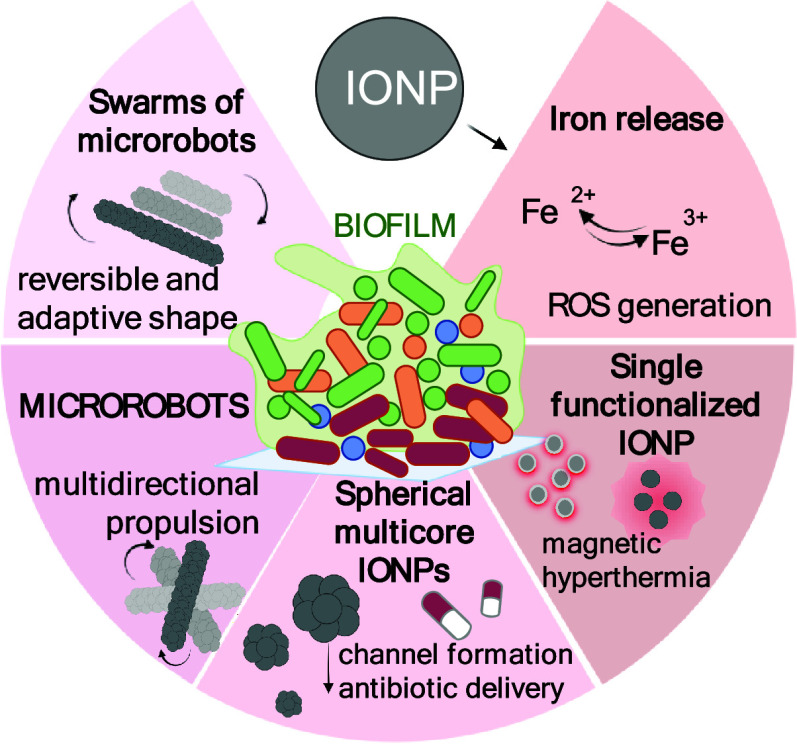

## 1 Introduction

Biofilms are dynamic structures formed by single or multiple species of microorganisms embedded in a self-produced matrix, also known as the extracellular polymeric substances (EPS) matrix.^1^ This matrix consists of polysaccharides, proteins, extracellular DNA, lipids, humic substances, membrane vesicles, enzymes, lipopolysaccharides, and phospholipids.^2,3^ Biofilms play a crucial role in the survival and persistence of bacteria and contribute significantly to antibiotic resistance. The EPS matrix acts as a physical and chemical barrier that limits the penetration, diffusion, and action of antibiotics.^4^ Within this protective environment, the microorganisms are thus protected from chemical, biological, and physical stress factors. In addition, bacteria in biofilms contain slow-growing, dormant, and metabolically less active or inactive cells, making them less susceptible to chemicals, including antibiotics, which generally target actively dividing cells. At the same time, horizontal gene transfer and the acquisition of resistance traits are promoted. Due to these factors, biofilm-associated infections are particularly difficult to eradicate.^5–7^

Harsh physical approaches, such as surface scraping, gamma irradiation, plasma-based technologies, ultrasound treatment, electric field-based methods, exposure to heat, or mechanical disruption, are currently used in industrial and medical settings to eradicate biofilms on contaminated extra-corporeal surfaces.^8^ Surface scraping and abrasion physically remove biofilms from contaminated materials. In a similar way, ultrasonic treatment, which uses high-frequency sound waves, has been employed to break apart biofilm structures and enhance the penetration of antimicrobial agents.^9^ Heat treatment, including high-temperature sterilization and localized thermal therapy, can denature the biofilm matrix and kill embedded bacteria.^10^ Plasma-based technologies, including cold atmospheric plasma, generate reactive species that degrade biofilms and kill bacteria without excessive heat damage.^11^ Similarly, high-speed and high-pressure processing, commonly used in food industries, disrupts biofilm integrity by applying extreme forces to bacterial aggregates.^12,13^

However, while relatively efficient for contaminated surfaces, the mentioned physical methods cannot be applied generally to biofilms within living tissues or implantable medical devices due to their destructive nature and potential for tissue damage. In clinical settings, biofilms forming on implanted medical devices, such as catheters, prosthetic joints, and pacemakers, pose significant challenges, as removal of the device is often required when biofilm-associated infections become untreatable with antibiotics alone.^14,15^ Additionally, biofilms in chronic wounds, lung infections (such as in cystic fibrosis patients), and other host-associated environments are particularly difficult to target with purely physical methods.^16,17^ On the contrary, current therapeutic strategies of treating biofilm-associated infections in patients often rely on combining multiple antibiotics, increasing dosage, and/or extending the duration of antibiotic treatment. Consequently, this approach not only often fails to fully eradicate the biofilm but also provides the perfect ground for bacteria to refine their resistance strategies, ensuring that future infections become even more untreatable.^18^

To address these challenges, alternative methods emerged, which aim to tackle biofilms using multifaceted strategies, by combining physical approaches with chemical or biological treatments.^19^ For example, low-intensity ultrasound combined with antibiotics can enhance drug penetration into biofilms, while weak electrical stimulation has been shown to increase antibiotic efficacy and disrupt bacterial communication (quorum sensing) within biofilms.^9,20^ Cold plasma treatment has demonstrated potential for wound healing applications by eradicating biofilms while promoting tissue regeneration.^21,22^ Electric field-based methods, such as high-intensity pulsed electric fields (PEFs) combined with antibiotics, enhance the penetration of antibiotics in the biofilms, leading to biofilm disruption and bacterial eradication.^23^ Nevertheless, this effect cannot be generalized to all biofilms and antimicrobial agents.^24^ Bacteriophages (bacterial viruses) are nature’s best-known bacterial opponents and have been used as antibiotic alternatives in many countries, including the former Soviet Union, Central Europe, France, and Brazil, and are currently considered an emerging component of personalized medicine for the treatment of antibiotic-resistant bacteria.^25,26^ Alternatively, probiotic and commensal bacteria or, more recently, immunotherapy has been explored as potential biofilm-targeting strategies.^27,28^

Nanotechnology offers innovative tools for multimodal platform design, which could even further enhance the effectiveness of biofilm eradication. Among these, inorganic and particularly magnetic iron oxide nanoparticles (IONPs) (Figure 1 and Supporting Information Table S1) have emerged as powerful agents due to their tunable properties, including superparamagnetism, surface functionalization, and controlled targeting via external magnetic fields.^4,29^ IONPs can disrupt biofilms through multiple mechanisms, such as reactive oxygen species (ROS) generation, enhanced antimicrobial delivery, and mechanical disruption of the EPS matrix. By leveraging these properties, IONPs offer a promising strategy to overcome the limitations of conventional antibiotics.^30,31^

**Figure 1:**
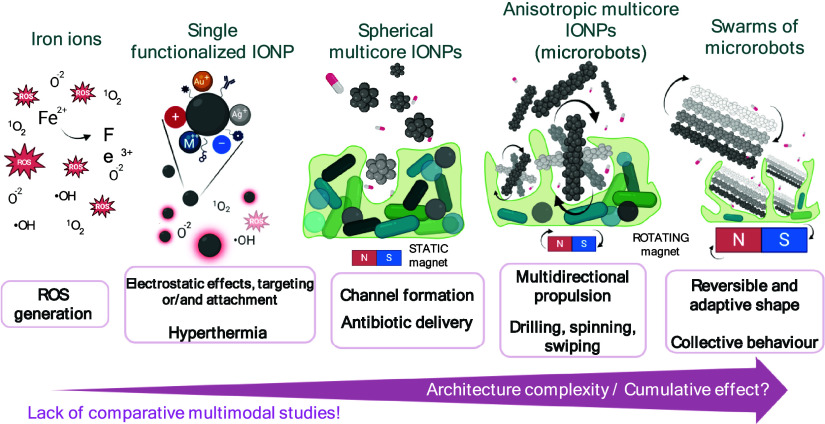
Schematic overview of the developmental stages of IONP nanosystems, progressing from released iron ions and single-crystal IONP cores to complex magnetic microrobot swarms for antibiofilm and antibacterial applications. The advancement and diversity of treatment modalities increase in parallel with the structural complexity and design sophistication of the nanostructures and their assemblies. Created with BioRender.

Nanotechnology-driven biofilm control is an emerging field with significant potential. In this review, we examine the resistance mechanisms that make biofilms difficult to eradicate, highlight recent advances in the use of IONPs in preclinical settings, and discuss the benefits and limitations of individual IONPs or their multicore assemblies for biofilm disruption. By integrating IONPs with antimicrobial strategies, more effective biofilm management solutions can be developed, ultimately improving patient treatment outcomes and enhancing industrial applications.

## 2 Biofilm Structure and Function

Biofilms (Figure 2) are microbial communities enclosed in a self-produced hydrogel-like matrix of extracellular polymeric substances (EPS) that provides numerous benefits, such as physical protection from the host’s immune system and environmental factors (e.g., UV light, acids, salinity, antibiotics, disinfectants, and detergents). The matrix also enables the retention of water, the storage of nutrients, and the exchange of genetic information, which promotes microbial persistence. In the biofilm, the matrix also provides the microbes with physical stability and resistance to mechanical removal, as the viscoelasticity associated with the matrix makes detachment difficult even under prolonged shear stress or high mechanical pressure.^2,6,32^ Biofilms on surfaces form different architectures, ranging from small and scattered clusters, small aggregates with variable thicknesses, mushroom-shaped structures of different sizes, flat and compact structures, or patchy coverage to confluent growth with clump formation. In addition, there is a marked variability in three-dimensional biofilm architectures between different species and even between strains of the same species.^33^ Variations in the biofilm architecture depend on growth conditions, growth medium, and species composition; therefore, different structures can be found in distinct environments, ranging from thick and layered microbial mats to thin and small aggregates.^33,34^ The thickness of biofilms can range from biofilms with a single cell layer less than 5 μm thick to microbial biofilms with mixed species that are 1000 μm thick, as measured for *Pseudomonas aeruginosa* (*P. aeruginosa*) and *Klebsiella pneumoniae* (*K. pneumoniae*). This depends on (i) species composition, with thicker biofilms having greater microbial diversity compared to thinner biofilms, and on (ii) nutrient availability, with high nutrient concentrations leading to thicker biofilms.^35–37^ Microbial biofilms form not only on surfaces but also as aggregates in liquids by clonal growth, coaggregation, or aggregation induced by bacterial EPS or host factors. Their size is generally between 5 and 200 μm, although examples of *P. aeruginosa* aggregates with a diameter of 5–400 μm have been observed, and pelagic aggregates typically range from 500 μm to centimeters in size. The bacteria themselves are typically 0.2–2 μm wide and up to 5 μm long.^34,38^ Both types of biofilms, surface-bound and suspended aggregates, are characterized by a dynamic heterogeneity of subpopulations and a physiological stratification in space and time.^2,34^

**Figure 2:**
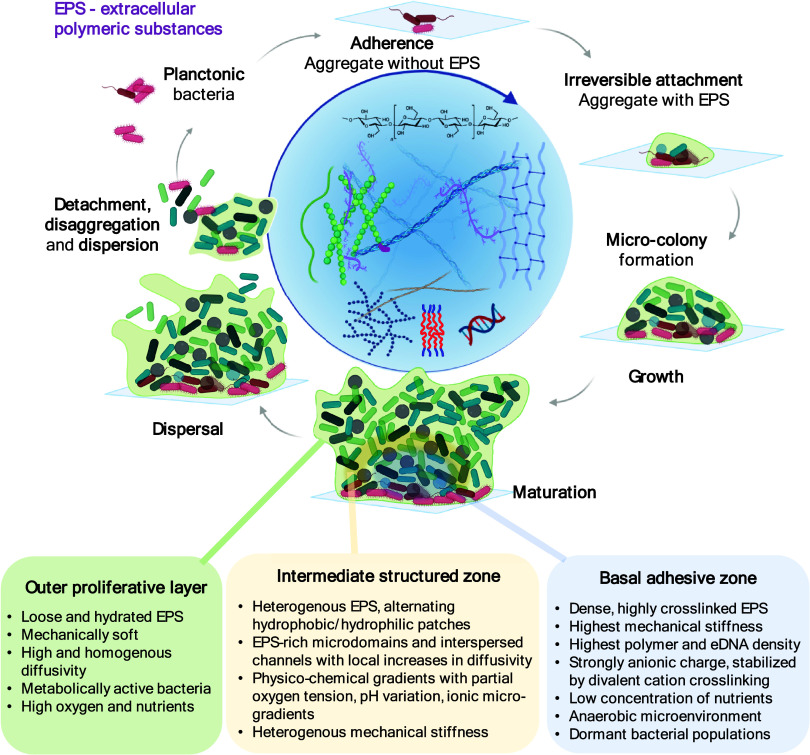
Biofilm formation begins with the initial reversible attachment of bacterial cells to a surface, followed by irreversible attachment, microcolony development, and maturation into a structured biofilm enclosed in a self-produced hydrogel-like matrix of extracellular polymeric substances (EPS). Ultimately, dispersal of individual cells or multicellular aggregates enables colonization of new surfaces. The model proposed that the formation of biofilms is a cyclic process that occurs in a multistage-specific and progressive manner. A mature biofilm consists of multiple physically, chemically, and biologically distinctive layers. This complexity has a direct impact on IONP penetration, magnetic actuation efficiency, and therapeutic performance. Created with BioRender.

The EPS matrix of biofilms consists of exopolysaccharides, nucleic acids (both extracellular DNA and RNA), proteins, lipids, and other biomolecules (Figure 2 and Supporting Information Table S2).^1^ These components have protective properties that need to be considered when developing biofilm removal strategies. Exopolysaccharides are an important component of the matrix and are very diverse. They have important functions in adhesion, scaffold formation, stability, cohesion, cell–cell binding, immune evasion, and protection against antimicrobial substances. Polysaccharides consist of repeating units of monosaccharides connected through rigid or flexible glycosidic linkages and branches. They either have a neutral charge or a negative charge provided by acidic sugars that make the polymers polyanionic. Polysaccharide complexity is further increased by substitution with noncarbohydrate organic or inorganic residues with various charges. Ester-linked acetyl residues and ketal-linked pyruvate, as well as sulfation and phosphorylation, are very common. These components influence the physical properties of the polysaccharide. For example, uronic acids and pyruvate have a polyanionic effect that affects solubility and ion binding, while acetylation and methylpentoses influence solubility, with the latter exhibiting increased lipophilicity. Depending on their structure, the exopolysaccharides can absorb and bind relatively large amounts of water, which is crucial for a good hydration state of the matrix and ultimately for the survival of the microbes in the biofilm. Different microbial species produce different polysaccharides, and even individual strains produce multiple polysaccharides at different stages of biofilm development and depending on changing environmental conditions. Cellulose is one of the most prevalent exopolysaccharides in the biofilm matrix, providing mechanical strength comparable to steel. Moreover, *P. aeruginosa*, for example, produces at least three polysaccharides: Psl (polysaccharide synthesis locus), Pel (pellicle), and alginate. Psl consists of pentameric units of glucose and mannose linked by β-1,3- and β-1,4-glycosidic bonds and carries a neutral charge. Pel is a cationic (positively charged) exopolysaccharide consisting of dimeric repeats of α-1,4-linked galactosamine and *N*-acetylgalactosamine forming a linear polymer. Alginate, on the other hand, is a linear polymer of β-1,4-linked mannuronic acid and guluronic acid residues, which gives it a negative charge.^39–43^ Polysaccharide intercellular adhesin (PIA) protects *Staphylococcus epidermidis* (*S. epidermidis*) against major components of the human innate immune system.^44^ PIA is a homopolymer of *N*-acetylglucosamine residues linked via β-1,6-glycosidic bonds. Its cationic nature allows electrostatic interaction with negatively charged molecules at physiological pH, such as extracellular DNA, other negatively charged polysaccharides, and host cell membranes. The functional relevance of its positive charge lies in its ability to promote intercellular adhesion in biofilms and contribute to overall biofilm structural integrity.

Proteins in the EPS also play a crucial role in maintaining structural integrity, adhesion, matrix modification, and metabolism. The enzymes are important for (i) degradation of polymers to obtain carbon and energy sources for microbial metabolism as an external digestive system; (ii) matrix remodeling through the activities of hydrolases, esterases, proteases, and lyases, which enable continuous restructuring of the EPS matrix; and for (iii) detachment and dispersion of biofilms. Another group of proteins found in the EPS are amyloid fibrils that are formed by self-assembling proteins adopting a highly ordered, water-insoluble structure of noncovalently linked β-sheets. They contribute to the maintenance of biofilm homeostasis by stabilizing the biofilm against mechanical, thermal, and chemical stress factors. They are also involved in cell division, nutrient storage, cell communication, adhesion, virulence, and evasion of the immune system. Examples of amyloids are curli in *Escherichia coli* (*E. coli*) and the TasA protein in *Bacillus subtilis* (*B. subtilis*). In addition, cell surface-associated proteins, such as lectins or adhesins, protein subunits of pili and fimbrae, and proteins released within extracellular vesicles contribute to adhesion, cohesion, and stability of the EPS matrix as well as to the regulation of growth and cell–cell signaling. Proteins in the EPS matrix are often positively charged, which enables their interaction with the negatively charged cell surfaces and extracellular DNA and contributes to the structural stability of the biofilm. In addition, proteins may also contribute to the formation of hydrophobic regions within the highly hydrated EPS matrix. For example, hydrophobin BslA proteins in *B. subtilis* form a hydrophobic film that makes the surface of the biofilm water-repellent.^1,41,45^

Extracellular DNA is an integral component of the EPS matrix with negative charge and provides (i) structural integrity through interactions with other biopolymers, i.e., polysaccharides and proteins; (ii) protection from antimicrobial agents through chelation of cations, including divalent cations and cationic antimicrobial peptides; (iii) a source of organic carbon, nitrogen, and phosphate; and (iv) a template for gene transfer and DNA damage repair. It is also essential for the initial adhesion and assembly of the biofilm matrix. Through interactions with polysaccharides and proteins, DNA provides the viscoelastic properties to biofilms and forms an interconnected network that increases the stiffness of the biofilm and its resistance to mechanical forces on the one hand and its fluidity and mechanical strength on the other.^40,46^

In addition to the main macromolecules mentioned above, the EPS matrix contains various other molecules that functionally contribute to the incredible adaptability of the EPS matrix and include both microbe-derived molecules (e.g., lipids, extracellular RNA, lipopolysaccharides, metals, and extracellular membrane vesicles) and molecules from the environment (e.g., host-derived polymers, humic substances, and bacteriophages).^40,41^

The EPS matrix provides important structural and functional properties for the creation of a heterogeneous microenvironment within the biofilm (Figure 2), allowing for the stratification of species composition and the development of dynamic nutrient and chemical gradients, including oxygen, pH, signaling molecules, inorganic ions, and metabolites, to promote microbial survival therein. The cohesion of the matrix is based on four types of noncovalent bonds (hydrogen bonds, ionic bonds, electrostatic interactions, and hydrophobic interactions), affecting the entanglement of the biopolymers and the water content regulated by the polymer density. The physical stability and resilience are due to the viscoelastic nature of the biofilm, which exhibits incredible variability in the degree of elasticity (how much it can stretch and spring back) and viscosity (how much it can flow under mechanical stress), so that the rheological behavior of the biofilm can range from resemblance to elastic solids to low-viscosity liquids.^34,40,41^ The dynamic and highly variable and adaptive nature of the EPS matrix is essential for the emergent properties of biofilm persistence and poses a challenge for biofilm prevention and removal approaches.

Biofilm removal requires eliminating both bacterial cells and their EPS matrix. Biofilm reduction is often reported as a percentage, especially when both the matrix and bacteria are detected using methods such as the crystal violet assay. However, determining the number of bacteria (e.g., colony-forming units, CFU) provides a more precise measure of microbial reduction using the logarithmic scale, where a 1-log_10_ reduction corresponds to a 90% decrease in viable organisms, a 2-log_10_ reduction to 99%, a 3-log_10_ reduction to 99.9%, and so on. In the context of antimicrobial efficacy, disinfection typically requires a 3- to 5-log reduction depending on the application, while sterilization requires at least a 6-log_10_ reduction to ensure complete elimination of bacteria, including spores. To standardize and facilitate comparison across studies in this review, inhibition percentages from studies based on viable bacteria counts or CFU can be converted to log_10_ reduction values using the formula log_10_ reduction = −log_10_(1 – (% inhibition/100)), while crystal violet assay results cannot be converted this way.^47^

## 3 Role of Single-Crystal (Single-Core) IONPs in Biofilm Management

Nanomaterials have long been a significant focus of research within the scientific community due to their exceptional properties, including increased reactivity, large surface-to-volume ratio, cost-effective production methodologies, and robust chemical and thermal stability.^19,48^ These features position nanomaterials as versatile entities with vast potential, particularly in the biomedical field where their physicochemical properties can be precisely adjusted for therapeutic advancements.^49–52^ The recent applications of different nanomaterials in biofilm-targeting approaches mark a paradigm shift in microbial infection control.^53^ The targeted delivery of conventional antimicrobial agents offers a potent treatment option against a spectrum of pathogens, presenting a remarkable potential to enhance the efficacy of current strategies, while minimizing collateral damage to surrounding tissues.^19,48,54,55^

A broad array of nanoparticles has been suggested in preclinical settings, comprising but not restricted to metallic nanoparticles (silver, gold, and copper), metal oxide nanoparticles (iron oxide, titania, zinc oxide, manganese cobalt ferrite, and silica), polymeric nanoparticles (chitosan, polyethylene glycol, and polylactic-*co*-glycolic acid), lipid-based nanoparticles (liposomes and solid lipid nanoparticles), carbon-based nanoparticles (graphene and carbon nanotubes), and quantum dots.^56–80^

Among listed nanoparticle types, IONPs (Figure 3) offer an unparalleled set of properties, including magnetism, which allows remotely controlled guidance of magnetic nanostructures, contrast generation in magnetic resonance imaging,^81^ magnetic particle imaging,^82^ heat generation in magnetic hyperthermia, and targeted drug and gene delivery.^48,83–86^ Importantly, the iron oxides are biocompatible and generally recognized as safe (GRAS) by the Food and Drug Administration (FDA) agency, while, depending on their chemical composition (i.e., presence of Fe^2+^ and Fe^3+^), they could also be involved in ROS generation via Fenton reaction^87,88^ (Supporting Information Table S1).

**Figure 3:**
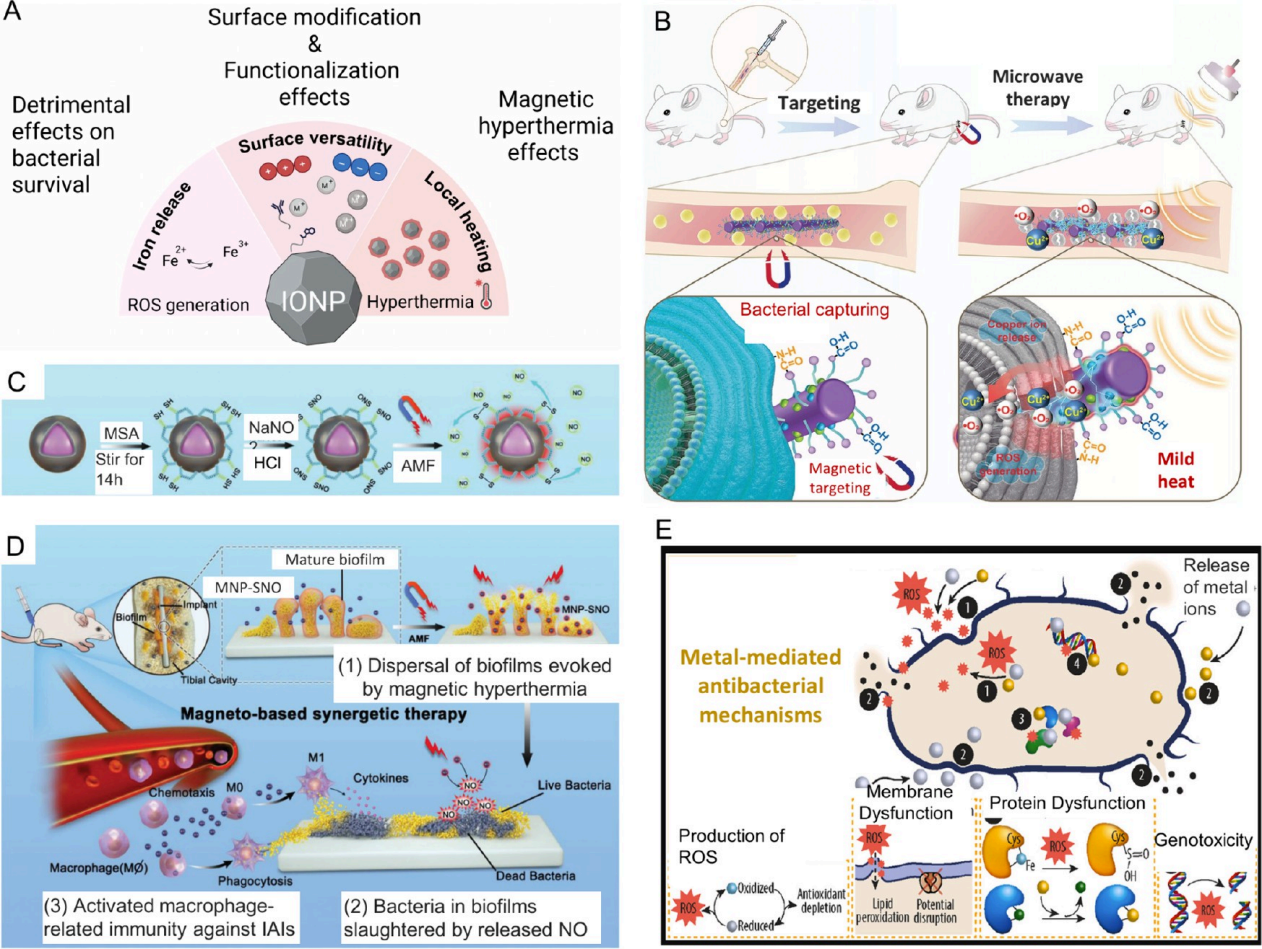
Schematic representation of the potential of single-core IONPs as biofilm-disrupting agents. Mechanisms include ROS generation, magnetic hyperthermia, co-delivery of toxic metal ions, and tailored surface modifications—such as controlled surface charge (positive or negative), hydrophobicity, inorganic or polymeric coatings, and attachment of targeting ligands (A). Created with BioRender. Cu-IONP-COOH captured bacteria by anchoring to amino groups on the surface of *S. aureus*. Magnetic targeting further contributed to preventing systemic bacterial dissemination in the treatment of *S. aureus*-induced osteomyelitis (B). Reprinted with permission from ref 89. Copyright 2024 Wiley-VCH GmbH. Schematics illustrating the ligand exchange and nitrosation processes of magnetic Co/Mn-IONPs, along with simultaneous magnetic hyperthermia and nitric oxide (NO) gas generation under an applied alternating magnetic field (AMF) (C). Reproduced from ref 90. Available under CC-BY 4.0. Copyright 2021 Wiley-VCH GmbH. Schematics illustrating a rat tibia with an infected implant under treatment by nanoparticles under AMF (D). Reproduced from ref 90. Available under CC-BY 4.0. Copyright 2021 Wiley-VCH GmbH. Antibacterial mechanisms of metal ions and nanoparticles, which can be entrapped in or coated on IONPs (E). Adapted from ref 91. Available under CC-BY 4.0. Copyright 2025 American Chemical Society.

### 3.1 IONPs as Potential Antibacterial and Antibiofilm Agents

Studies show that IONPs possess (i) intrinsic antibacterial properties, primarily due to their ability to generate ROS;^92–94^ (ii) enhanced effectiveness due to their facile functionalization (particularly positively charged and hydrophobic surfaces);^95–98^ (iii) improved contact interactions with bacterial membranes and biofilm components leading to stronger antibacterial effects;^99–101^ and (iv) synergistic effects when combined with H_2_O_2_ to further boost ROS production, resulting in significantly improved antibacterial activity.^102–105^ Furthermore, IONPs have also been utilized to cross-link and immobilize enzymes, enhancing their efficiency in biofilm eradication.^106,107^ These approaches highlight IONPs’ versatility in addressing persistent biofilms (Supporting Information Table S3).

#### 3.1.1 IONP Crystal Core-Related Effects: The Influence of Released Iron Ions

Iron ions are essential nutrients for bacterial growth (cofactors of enzymes) but also a toxic agent due to their role in ROS generation. The ROS are typically byproducts of incomplete oxygen reduction and are controlled by the cell’s antioxidant defense systems. Magnetic iron oxide nanoparticles (Figure 1) most commonly consist of magnetite (Fe_3_O_4_) and its oxidized form of maghemite (γ-Fe_2_O_3_). Both are iron oxide minerals with the same crystal structure, but they differ in chemical composition, namely, the oxidation state of iron. Magnetite is composed of Fe^2+^ and Fe^3+^ ions, while maghemite is composed of Fe^3+^ ions only. The composition is therefore crucial to understanding the intrinsic antimicrobial properties of IONPs, which might derive from the released iron ions. Under aerobic conditions and neutral pH, iron predominantly exists in its nearly water-insoluble Fe^3+^ form. Conversely, Fe^2+^ ions are much more soluble and available under anaerobic or acidic conditions. Based on the hard–soft acid–base theory, which classifies Fe^3+^ species as a hard acid and Fe^2+^ species as a borderline acid, Fe^3+^ preferentially binds to hard bases such as oxygen-containing ligands (e.g., hydroxyl, carboxyl, and phosphate), while Fe^2+^ tends to preferentially interact with softer ligands such as nitrogen (e.g., in porphyrins like heme) and sulfur (e.g., in iron–sulfur clusters).^92^ In general, iron ion availability and ligand preferences have significant implications for bacterial survival, metabolism, and pathogenesis.^108^

The basis of iron toxicity relies on two main mechanisms. The first is the catalytic activity of Fe^2+^ ions, which induces the generation of ROS via the Fenton reaction, resulting in the production of hydroxyl radicals, which can damage bacterial DNA, proteins, and components of membranes and the bacterial cell wall. The second mode of action involves the iron ion overload toxicity, where excessive Fe^2+^ ions interact with and inactivate bacterial enzymes and interfere with bacterial metabolism. In addition, beyond the direct action on bacteria, a reciprocal influence between iron ions and antibiotics has been suggested and is concisely reviewed elsewhere.^109^

The antibacterial effect of IONPs was determined in different studies. Al-Shabib et al.^110^ prepared IONPs of 6–9 nm by a low-temperature solution route using ferric chloride and ferric sulfate, precipitated by ammonia and stabilized by polyethylene glycol (PEG), which were tested on *E. coli* (ATCC 25922), *P. aeruginosa* (PAO1), *Serratia marcescens* (*S. marcescens*, ATCC 13880), and *Listeria monocytogenes* (*L. monocytogenes*,lab isolate). The nanoparticles showed MIC values of 32–128 μg/mL (32 μg/mL for *S. marcescens* and *L. monocytogenes*; 64 and 128 μg/mL for *E. coli* and *P. aeruginosa*, respectively). At subinhibitory concentrations (1/16–1/2 × MIC), IONPs inhibited biofilm formation by *P. aeruginosa* (16–82%), *E. coli* (28–77%), *L. monocytogenes* (22–88%), and *S. marcescens* (19–75%) in a dose-dependent manner. The mechanism of action involved ROS generation via the Fenton reaction, leading to oxidative damage of bacterial cells.^110^ Similar studies using iron oxide-based nanoparticles reported ROS-mediated antibacterial activity, though differing in size, morphology, and target strains. Cubic SPIONs (8 nm) inhibited *S. epidermidis* biofilms at low doses,^111^ while spherical IONPs (10–120 nm) showed broad-spectrum effects mainly against Gram-positive bacteria.^112^ Smaller IONPs (6–9 nm) show stronger antibacterial effects, likely due to the enhanced specific surface area and concomitantly greater iron ion release and consequent higher ROS generation.

Beyond direct action of iron ions on bacteria^110–113^ and their influence on antibiotics,^109^ iron oxides can also be used to improve the bactericidal effect of macrophages, as indicated by Yu et al.^114^ Namely, IONPs were used to promote the polarization of macrophages into the M1 (proinflammatory subtype). Such IONP-exposed macrophages thus exhibited a stronger bactericidal effect when applied to wounds of mice infected with Staphylococcus aureus (*S. aureus*) and reduced the bacterial burden by 25% compared to the untreated control group.^114^

#### 3.1.2 IONP Surface Functionalization: The Influence of Polymers, Coatings, and Surface Charge

In addition to the IONPs’ core, the surface properties of the IONPs (Figure 3A) have a significant influence on the bactericidal effect. In a study comparing the toxicity of uncoated (bare IONPs, a slightly positive ζ potential of approximately 6 mV at physiological pH), carboxyl-functionalized IONPs (negatively charged; a ζ potential of approximately −20 mV at physiological pH), and amino-functionalized IONPs (positively charged; a ζ potential of approximately 22 mV at physiological pH) with a comparable diameter (approximately 20 nm), toxicity increased with concentration, while positively charged nanoparticles exhibited the highest bactericidal effect against biofilm-embedded *Streptococcus mutans* (*S. mutans*) (30% vs about 20% for bare and negatively charged nanoparticles). Nevertheless, over the incubation time (3 h), the ζ potential of all three types of nanoparticles started to converge due to the adsorption of biofilm components and formation of corona, shifting all values toward approximately −15 to −17 mV.^115^ Related studies point to clear evidence that surface chemistry critically shapes IONP–biofilm interactions. Positively charged APTES- and chitosan-coated IONPs significantly reduced biofilm formation through enhanced electrostatic interaction and ROS generation,^99,116^ while investigations on surface hydrophobicity showed that oleic acid- and rhamnolipid-functionalized IONPs effectively inhibited bacterial adhesion and disrupted preformed biofilms.^117,118^ Taken together, the evidence demonstrates that surface charge and hydrophobicity are critical determinants of IONP antibacterial and antibiofilm activity, primarily through their interactions with the negatively charged and partially hydrophobic biofilm matrix.^8,117,119,120^

Interestingly, instead of direct antibiofilm action, positively charged APTES-functionalized IONPs were used as a tool to magnetically cross-link polyphenol oxidase, resulting in enzyme aggregates that were tested for inhibition of microbial biofilm. Polyphenol oxidase aggregates showed up to 70–75% inhibition of *E. coli*, *S. aureus,* and *K. pneumoniae* biofilms with a decrease in carbohydrate and protein contents in the biofilm’s EPS.^107^ The aggregates could be reused up to five times with consistent efficiency in biofilm inhibition. Similarly, in another study by the same authors, IONPs were used to magnetically cross-link multiple enzymes (amylase, trypsin, cellulase, horseradish peroxidase, and a blend of pectinases, hemicellulases, and arabinanases) and observed 75–78% inhibition of *E. coli* and *S. aureus* biofilms.^106^ Furthermore, hydrogen peroxide (H_2_O_2_) and IONPs have been investigated as a potential synergistic dual system with enhanced antimicrobial efficacy.^104,105^ This approach harnesses the catalytic properties of IONPs to generate ROS, such as hydroxyl radicals, via Fenton-like reactions. This strategy underscores the potential of IONPs in combination with H_2_O_2_ as a potent antimicrobial treatment, offering a promising avenue for combating microbial infections, including those involving biofilms. The synergistic H_2_O_2_ and IONP action has been shown to degrade the dental biofilm (*S. mutans*) matrix and rapidly kill embedded bacteria with a 3-log_10_ reduction in 5 min.^105^ Moreover, dextran-coated IONPs exhibited robust peroxidase-like activity at acidic pH values and facilitated the penetration of particles into the EPS biofilm matrix, activating H_2_O_2_ and then enabling targeted biofilm disruption and caries prevention *in vivo*. In the latter study, dextran served as a colloidal stabilizing agent for IONPs and facilitated selective binding to biofilm components, enabling targeted bacterial removal and biofilm disruption, while avoiding damage to surrounding areas. Dextran-coated IONP formulations reduced bacterial biomass significantly more than H_2_O_2_ alone.^104^

#### 3.1.3 IONPs Decorated with Antibacterial Metal Ions and/or Nanoparticles

Recent studies present the combination of IONPs with various metal ions, such as silver, copper, gold, and magnesium, to synergistically enhance the antimicrobial efficacy of nanocomposites (Figure 3).^89^ Metal ion-decorated IONPs exhibit improved capabilities in reducing biofilm formation, leveraging the combined antimicrobial properties of the nanocomposite.^121^ Metallic nanoparticles interact with bacterial membranes via a range of mechanisms, including electrostatic attraction, van der Waals forces, receptor–ligand binding, and hydrophobic interactions. Upon attachment, these nanoparticles can penetrate biofilms and attach to bacterial cells to interfere with extra- and intracellular processes by inhibiting enzymatic activity, denaturing proteins, inducing oxidative stress, disrupting electrolyte balance, and altering gene expression, ultimately impairing critical metabolic pathways.^122^ The bacterial cell envelope plays a crucial role in bacterial defense. Its surface-exposed membrane proteins, typically bearing a negative charge at neutral pH, readily bind positively charged metal ions and nanoparticles. These interactions are often species-specific, reflecting differences in membrane composition and protein structure. Additionally, lipid components of the membrane, particularly phospholipids, can interact with metal ions or nanoparticles, leading to modifications in membrane charge, hydration state, and dipole potential. Such changes compromise bacterial membrane integrity and increase permeability. These interactions can initiate the formation of ROS and promote lipid peroxidation, resulting in reduced membrane fluidity and increased permeability. At elevated concentrations, the metallic nanoparticles may cause physical rupture of the membrane, leading to the leakage of cytoplasmic contents. In an attempt to counteract this stress, bacteria may enhance proton efflux and electron transport activities; however, such responses often exacerbate membrane damage and accelerate cell death. Once internalized, metal ions bind to reactive side chains of amino acids, including thiol groups in cysteine, carboxyl groups in aspartate and glutamate, and amine groups in lysine, interfering with protein structure and function. Given that many metal ions serve as essential cofactors for enzymatic processes, cells maintain strict regulation over metal ion homeostasis to prevent mismetalation and minimize ROS generation. In a similar way to iron, discussed in Section 3.1.1, other redox-active metals such as copper, chromium, and nickel ions can also catalyze Fenton reactions resulting in ROS (Figure 3E).^91^

Importantly, antibiofilm properties of Ag-decorated IONPs have been shown against methicillin-resistant *S. aureus* biofilms. In this study, planktonic bacteria growth was reduced from 88% to 58% at 1 mg/mL, while a lower 0.01 mg/mL concentration of Ag-decorated IONPs showed no significant effect. In this particular case, biofilm mass decreased by ∼30%.^123^ In another study, a nanocomposite composed of Ag nanoparticles and IONPs was applied against different microbial strains. The oxidative stress caused by ROS and the controlled release of the Ag ions led to good antibacterial activity of the nanocomposite with 5%, 10%, and 15% IONPs inhibiting *S. aureus* and *P. aeruginosa* biofilms by 7%, 10%, and 14–15%, respectively. Additionally, applying a magnetic field enhanced nanocomposite penetration, resulting in over 90% biofilm eradication; however, the authors did not provide details about the field strength, frequency, duration, or magnetic field generator type.^124^

Gold-decorated and amino-functionalized IONPs were investigated for their ability to limit the growth of bacteria *E. coli*, *S. aureus*, *P. aeruginosa*, and yeast *Candida albicans* (*C. albicans*). While the study was conducted on planktonic bacterial cells, the tested microorganisms are well-known biofilm formers, especially on medical devices; thus, the study is relevant for preventing early-stage biofilm formation. Pathogen capture was achieved by binding IONPs to bacteria in suspension, followed by magnetic separation and monitoring optical density (OD_600_). Bare IONPs showed the highest capture efficiency for *S. aureus* and *C. albicans*, while gold-decorated IONPs slightly improved capture of *P. aeruginosa*. Amino-functionalized IONPs exhibited lower capture potential but achieved significant growth inhibition, with rates of 75% (*E. coli*), 26% (*S. aureus*), and 99% (*P. aeruginosa*). These nanocomposites demonstrated effective pathogen removal from saline buffer and body fluids (∼100% efficiency) without causing significant red blood cell damage (<5% hemolysis).^125^ In an interesting study, magnesium ferrite (MgFe_2_O_4_) nanoparticles (25–35 nm) were investigated against Gram-negative *E. coli* and Gram-positive *S. aureus* and exhibited potent dose-dependent antibacterial toxicity. Minimum inhibitory concentrations were 1.25 μg/mL for *E. coli* and 2.5 μg/mL for *S. aureus,* while a higher concentration (10.0 μg/mL MgFe_2_O_4_ nanoparticles) elicited 89% and 78.5% reduction in *E. coli* and *S. aureus* biofilms, respectively.^126^

Finally, certain metal ions like aluminum, copper, and silver destabilize iron–sulfur clusters in essential metabolic enzymes, releasing free iron ions that further promote ROS production. Metal ions such as Ag^+^, Cd^2+^, and As^3+^ also contribute to oxidative stress by depleting intracellular levels of glutathione (GSH), a critical antioxidant molecule. The resulting accumulation of ROS leads to oxidative damage of bacterial lipids, proteins, and nucleic acids, playing a central role in the antimicrobial efficacy of metal ions and metal-based nanoparticles (Figure 3E).^91^

### 3.2 Exploitation of IONP-Based Magnetic Hyperthermia Effects

Magnetic hyperthermia (MHT) emerges as a promising technique utilizing the heat generated by IONPs when exposed to an alternating magnetic field (AMF) at relatively high frequencies (>100 kHz). This localized temperature increase is shown to be a potential strategy for eradicating bacterial biofilms due to enhanced penetration of therapeutic agents. Magnetic nanomaterials, particularly IONPs, have become central in hyperthermia research due to their unique heating response, chemical stability, easy functionalization, and biocompatibility.^97,127–130^ Additionally, the efficacy of IONPs in heat generation and damaging biofilms is dependent on various physicochemical properties, including particle size, shape, concentration, saturation magnetization, magnetic anisotropy, and experimental conditions such as the fluid type, frequency, and amplitude of the AMF.^131–134^ Numerous studies have been dedicated to exploring and summarizing MHT strategies, emphasizing the importance of tailoring IONPs for targeted and effective biofilm eradication (Supporting Information Table S4).^135–139^ One crucial aspect of IONP-assisted hyperthermia is appropriate nanoparticle targeting.^140–144^ The latter ensures that the heat generated by IONPs is directed specifically toward the biofilm or its specific components, minimizing off-target effects. This precision is vital for the clinical utility of magnetic hyperthermia in biofilm eradication. To demonstrate IONP targeting, a protein A antibody was attached to IONPs. This nanoscale system showed a 3-log_10_ reduction in *S. aureus* bioluminescence (∼99.9% bacteria killing) achieved at 502.8 Oe/40 kA/m and 2-log_10_ reduction (∼99% bacteria killing) at a higher IONP concentration and a lower magnetic field amplitude.^145^ This approach showed an 80% increase in *S. aureus* inactivation compared to the IONPs conjugated with nonspecific IgG, while the latter system showed a 50% increase in bacterial inactivation ability compared to the IONPs without an antibody. This study therefore confirms that specific contact interaction between IONPs and bacterium surface is required to potentiate the antibacterial effect of IONP-triggered magnetic hyperthermia.^145^

However, despite significant progress in the field, several challenges remain, including optimizing heating parameters, determining the ideal IONP concentration, and addressing issues related to particle size distribution, which can cause temperature gradients and toxicity. Additionally, targeting deep or multilayered biofilms, particularly in complex environments like chronic wounds or implanted devices, remains a significant challenge. Future research should focus on enhancing IONP delivery systems, refining *in vivo* application techniques, and exploring combination therapies with antibiotics or other antimicrobial agents to achieve more effective biofilm eradication.

#### 3.2.1 Magnetic Hyperthermia Effects of IONPs as Influenced by Their Coatings and Surface Functionalization

Magnetic hyperthermia using bare iron oxide nanoparticles (60 mg/mL) demonstrated potent antibiofilm activity against *P. aeruginosa*, achieving over a 4-log_10_ reduction in biofilm viability within 8 min under an alternating magnetic field (37.7 Oe/3 kA/m, 492 kHz), with the local temperature rising to 62.3 °C.^149^ Since bare IONPs frequently face colloidal instability in complex fluids, functionalized IONPs offer a viable alternative. For example, poly­(acrylic acid)-functionalized IONPs caused a significant decrease in cell viability (≥3-log_10_ CFU) in both planktonic and biofilm *Pseudomonas fluorescens*, with complete eradication of planktonic bacteria following 8 min of exposure to AMF (100 Oe, 7.96 kA/m, 873 kHz), increasing the temperature of the system to 55 °C.^150^ When comparing the same final temperatures, it was shown that magnetic hyperthermia heating was more harmful to the integrity of bacterial cell membranes than direct conventional heating. Although the mechanisms were not fully elucidated, one possible explanation is that conventional heating uniformly raises the bulk temperature, whereas magnetic hyperthermia generates localized hot spots close to nanoparticles. When these nanoparticles are in close proximity to bacterial cell membranes, the localized heating may induce greater membrane disruption and thus has a greater bactericidal effect. Furthermore, different IONP nanoscale architectures, such as mesoporous hollow IONPs (1 mg/mL), were studied under AMF conditions (2.5 kW, 210 kHz) and showed that the system could effectively eliminate 74% and 70% of *E. coli* and *S. aureus,* respectively, as the temperature was raised from 31 to 43 °C.^151^ The effect of the surface charge or ζ potential of IONPs on their ability to adhere to specific biofilm components when applying AFM was investigated by using bare and polyethylenimine-functionalized Fe_3_O_4_ (IONPs-PEI).^146^ It was reported that the number of planktonic bacteria remained almost unchanged when exposed to different concentrations of IONPs under an AMF activation. However, IONPs-PEI reduced *S. aureus* and *E. coli* biofilm biomass by 87.4% and 84.9%, respectively, which was related to the strong self-association ability of these positively charged nanoparticles that can electrostatically interact with oppositely charged, planktonic and sessile bacteria. IONPs-PEI nanoparticles produced physical stress and thermal damage in response to AMF, inducing the accumulation of ROS, resulting in bacterial membrane damage and biofilm loosening and dispersion (Figure 4A).^146^

**Figure 4:**
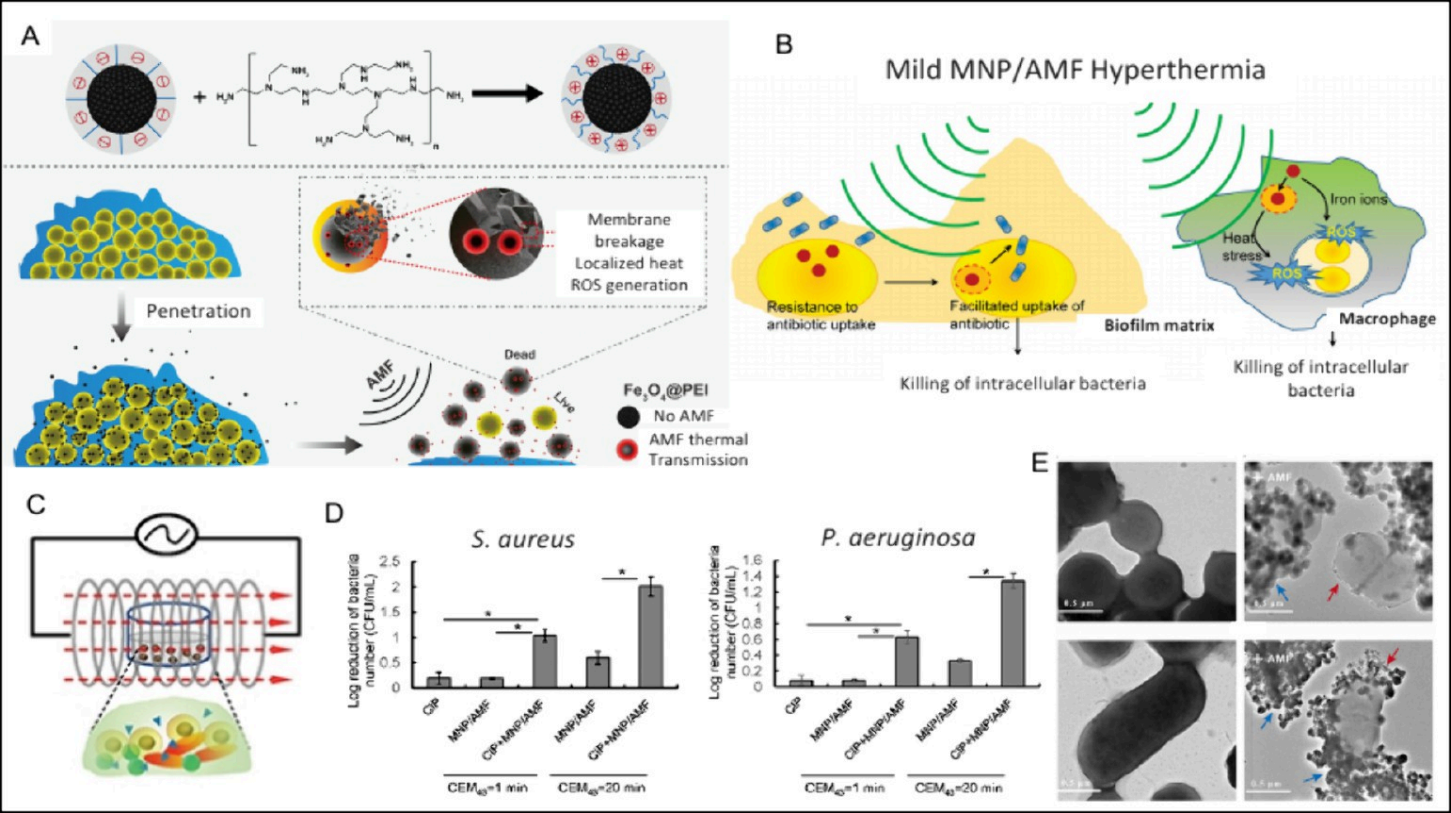
Schematic illustration of the underlying antibiofilm mechanism of IONPs activated by an AMF to induce magnetic hyperthermia. Heat generation by positively charged IONPs (A) (Reprinted with permission from ref 146, Copyright 2022 American Chemical Society), mild magnetic hyperthermia sensitizes *S. aureus* biofilm to antibiotics by facilitating their uptake into bacterial cells (B) (Reprinted from ref 147, available under CC-BY 4.0, Copyright 2020 Taylor & Francis Group). Experimental setup for magnetic hyperthermia treatment of a dual-species biofilm composed of *S. aureus* and *P. aerruginosa* (C). Reproduced from ref 148. Available under CC-BY-NC 4.0. Copyright 2023 Taylor & Francis Group. Effects of hyperthermia alone or in combination with ciprofloxacin on biofilm reduction (D). Reproduced from ref 148. Available under CC-BY-NC 4.0. Copyright 2023 Taylor & Francis Group. Transmission electron microscopy (TEM) images of *S. aureus* as control under control conditions and following AMF exposure (E). Adapted with permission from ref 146. Copyright 2022 American Chemical Society.

#### 3.2.2 Magnetic Hyperthermia in Combination with Antibiotics

Recent studies have also explored the synergistic potential of combining magnetic hyperthermia with antibiotics to enhance antibacterial efficacy (Supporting Information Table S4). This approach aims to improve thermostable antibiotic penetration into biofilms and infected tissues, thereby increasing bacterial eradication. Hyperthermia not only weakens bacterial cell walls but also loosens and disrupts biofilm matrices, rendering bacteria more vulnerable to antibiotic treatment.^152^ These combined effects highlight a promising strategy for enhancing the effectiveness of antibacterial therapies in combating infections. The effect of the combination of hyperthermia and an antibiotic as shown by a combination of 2 mg/mL IONPs and AMF (377.1 Oe, 30 kA/m, 2.1 MHz, 6 min), followed by the exposure to ciprofloxacin at 16 μg/mL, resulted in a 2-log_10_ reduction of the *S. aureus* biofilm, while the antibiotic alone applied at the same concentration had no effect and also a higher dose (up to 1024 μg/mL) resulted in only 1-log_10_ reduction. Similarly, an exposure to IONPs alone, under the same AMF conditions and antibiotic, resulted in a less than 1-log_10_ reduction. The authors attributed the observed synergistic effect to enhanced antibiotic uptake by biofilm-embedded bacteria. In addition, the application of hyperthermia could promote the bactericidal activity of macrophages against intracellular bacteria via IONP-dependent generation of ROS (Figure 4B).^147^ Related studies point to clear synergy between magnetic heating and antimicrobials: AMF-activated IONPs used with ciprofloxacin improved clearing of mixed-species biofilms and infected wounds over heat alone,^148^ polymer-functionalized IONPs dispersed the biofilm via mild AMF heating and markedly boosted gentamicin activity,^152^ and nitrosothiol-loaded CoFe_2_O_4_/MnFe_2_O_4_ IONPs used under AMF opened channels and rapidly released nitric oxide to kill dormant cells via ROS.^90^

## 4 Spherical Multicore IONP Assemblies

Although single-crystal core IONPs paved the way for introducing new nanotechnological approaches to combat bacterial biofilms, they are not the best candidates for magnetic targeting (Figure 5 and Supporting Information Table S1). Individual superparamagnetic IONPs experience only minimal magnetic force when exposed to magnetic field gradients due to their small volume and magnetic moment, making them ineffective for *in vivo* magnetic targeting without prior clustering into larger assemblies.^153^ To advance the use of nanoscale objects for mechanical loosening or supportive mechanical disruption of biofilms, magnetic IONPs must respond to external magnetic field gradients quickly and strongly enough to break at least the weakest points within the biofilm matrix structure.^29^ In this context, individual superparamagnetic IONPs completely fail, as the magnetic force exerted on single superparamagnetic IONPs in magnetic field gradients is not strong enough to move individual IONPs spatially in a diluted liquid like water, and even less so in complex, interbranched systems such as biofilms. There is a need to apply larger IONPs, as the magnetic force exerted on nanoparticles depends on their magnetic moment and, consequently on their volume. However, another problem arises: superparamagnetic IONPs quickly lose their superparamagnetism at room temperature once their nanocrystal size reaches approximately 20 nm, the size at which they become ferrimagnetic. This means that they behave as tiny magnets and thus tend to magnetically aggregate into larger, shapeless aggregates even without exposure to an external magnetic field. Such clumping is evidently unsuitable for the therapy. An alternative approach to increasing the particle volume while preserving the superparamagnetic state is the directed assembly of multiple individual IONPs into a larger multicore cluster, with a minimum cluster size of around 100 nm to enable efficient spatial guidance and maneuverability under a magnetic field gradient (Figure 6A).^29,154^ Such well-defined superparamagnetic IONP clusters, as well as to some extent also randomly aggregated multiple IONPs with broad size and shape distributions, could potentially be applied as remotely controlled nanoscale objects able to mechanically dig into or penetrate the biofilm matrix, creating unidirectional channels along their movement toward areas of higher magnetic field within a generated magnetic field gradient.^155^ Due to their ability to be magnetically guided, these spherical multicore IONP nanostructures create pores within the biofilm and facilitate access to sequentially administered antibiotics, or are suitable for delivering surface-bound antibiotics or other antimicrobial agents deep into the biofilm matrix, which is otherwise almost unreachable within the treatment time frame by simple diffusion. Numerous studies have demonstrated the efficacy of multicore IONP-based systems in delivering antibiotics like gentamicin, ciprofloxacin, and other antimicrobial compounds (Supporting Information Table S4). However, further development and optimization of multicore IONPs assemblies are needed, particularly in establishing a suitable compartment for drug loading, to improve the colloidal stability and effective remotely controlled release of carrying agents.

**Figure 5:**
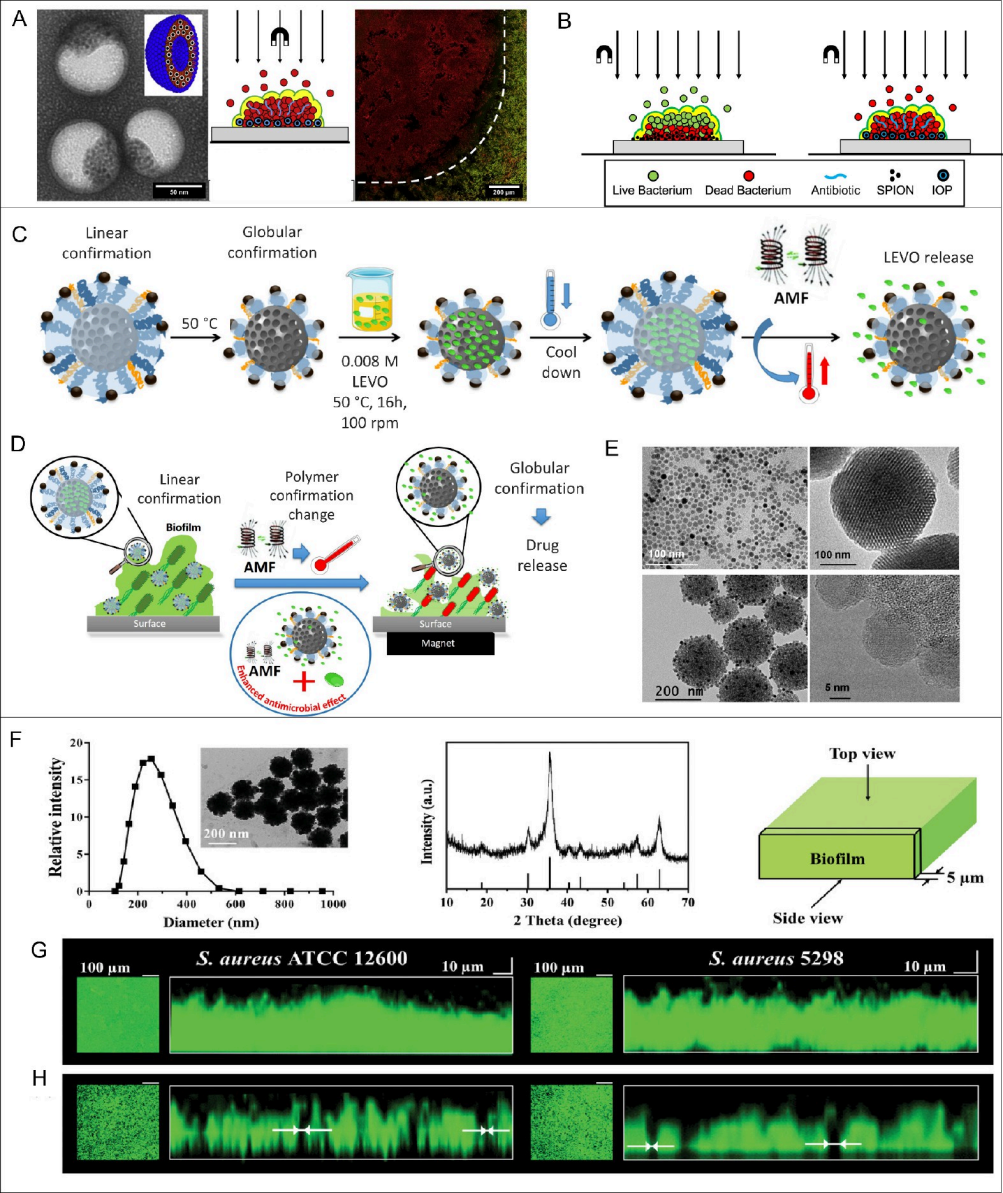
Current strategies for biofilm treatment using spherical multicore IONPs. Representative TEM of hydrophobic IONPs embedded inside the polymersome bilayer (A). Reprinted with permission from ref 156. Copyright 2016 Elsevier Ltd. Schematic representation of magnetic targeting and channel formation inside biofilm for easier antibiotic penetration (B). Reprinted with permission from ref 156. Copyright 2016 Elsevier Ltd. Schematic depiction of the experimental procedures for the loading and release of levofloxacin (LEVO) from the thermo-responsive multicore IONPs nanosystem (C). Dual mode of action for bactericidal effects generation by IONP/AFM heating and channels digging due to unidirectional movement of polymersome-packed multicore IONPs (D). TEM images of IONPs, bare porous silica particle, IONP-loaded thermo-responsive nanosystem, and its high-magnification image (E). Figures 5C−E reproduced from ref 157. Available under CC-BY 4.0. Copyright 2022 MDPI. Characteristics of spherical multicore IONPs and schematics of the procedure applied to obtain overlayer and transverse cross-sectional images of a biofilm in confocal laser scanning microscopy (CLSM) (F). Overlayer and transverse cross-sectional CLSM images of 24 h old S. aureus ATCC 12600 and *S. aureus* 5298 biofilms prior to artificial channel digging by multicore IONPs (G). Same as panel (G), but after digging artificial channels by moving multicore IONPs (H). Channels perpendicular to the substratum surface appeared as black dots on the green-fluorescent biofilms. Channel widths were measured in cross-sectional images, as indicated by white arrows. Figures 5F−G reproduced from ref 155. Available under CC-BY 4.0. Copyright 2019 John Wiley & Sons, Inc.

**Figure 6:**
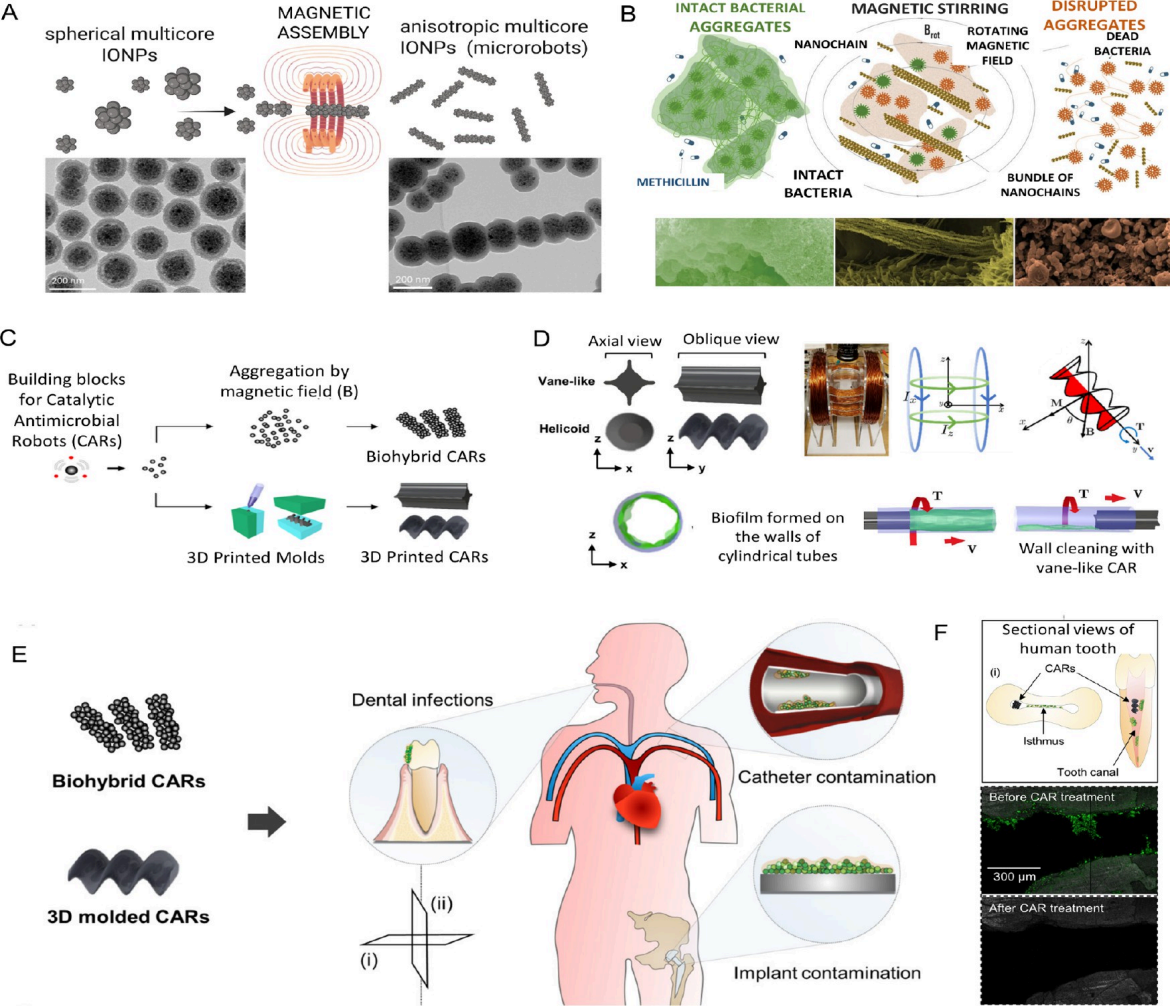
Current strategies for biofilm treatment utilizing multicore IONPs with anisotropic shapes—magnetic nano- and microrobots. Schematic illustration and corresponding TEM images showing the magnetic alignment and silica fixation of multicore IONPs into chain-like assemblies (nanorobots) (A). Adapted from ref 173 (Available under CC-BY 3.0, copyright 2023 The Royal Society of Chemistry) and with permission from ref 154 (copyright 2024 Wiley-VCH GmbH). Schematic representation of key stages in magneto-mechanical actuation: exposure of biofilm-forming bacterial aggregates to antibiotics and magnetic nanorobots leads to aggregate disruption, bactericidal effects, and prevention of further biofilm development (B). Reprinted with permission from ref 29. Copyright 2024 Wiley-VCH GmbH. Catalytic-IONPs served as multifunctional building blocks for the formation of catalytic antimicrobial robots (CARs). In the first platform, biohybrid CARs with bristle-like architectures were magnetically assembled from IONPs using a permanent magnet mounted on a micromanipulator, enabling efficient biofilm removal from accessible surfaces. In the second platform, catalytic-IONPs were embedded within hydrogels to create 3D-molded CARs featuring specialized vane- and helicoid-shaped structures for targeted mechanical disruption and antibacterial activity (C). Reprinted with permission from ref 174. Copyright 2019 Science Robotics. Model representations of vane-shaped and helicoid-shaped CARs fabricated via 3D micromolding of agarose gel embedded with IONPs. Both robot designs measure 5 mm in diameter and 10 mm in length. A Helmholtz coil system was employed to actuate the 3D-molded CARs within cylindrical tubes by applying sinusoidal, time-varying currents to each coil pair, generating uniform rotating magnetic fields. The CARs were operated at 3.4 mT and 4 Hz (D). Reprinted with permission from ref 174. Copyright 2019 Science Robotics. These robots are applicable for biofilm removal from both biotic surfaces (e.g., teeth) and abiotic substrates (e.g., catheters, implants). Their ability to access anatomically constrained regions was demonstrated by targeting the interior of human teeth (E). Reprinted with permission from ref 174. Copyright 2019 Science Robotics. Biohybrid CARs successfully navigated and reached the isthmus, emphasizing their potential for treating infections in complex anatomical environments (F). Reprinted with permission from ref 174. Copyright 2019 Science Robotics.

### 4.1 Spherical Multicore IONP Assemblies as Channel-Forming Magnetic Delivery Systems

Selected studies on the magnetic delivery of antibacterial agents are presented here, regardless of whether they employ well-defined assemblies of multiple IONPs or (un)­intentionally formed random aggregates of IONPs, both of which have varying degrees of ability to deliver antimicrobials to the magnetically targeted area (Supporting Information Table S5). In general, the controlled delivery of therapeutic compounds has been a key focus in nanomedicine, and among various drug nanocarriers, multicore IONP-based nanostructures stand out due to their exceptional magnetic and biological properties, which enable high drug loading capacity and magnetic targeting.^158^ Such a magnetic targeting approach not only enhances the therapeutic efficacy of antibacterial agents but also has the potential to minimize the systemic side effects typically associated with conventional antibiotic treatments. Moreover, the relatively small size and high surface area-to-volume ratio of IONPs facilitate their interaction with bacterial cells and biofilms, improving the penetration and retention of antibacterial agents at the site of interest.

An interesting study reported the use of 60 nm carboxyl-functionalized IONPs conjugated with gentamicin via peptide coupling. These gentamicin-loaded nanoparticles (25% w/w), most likely randomly magnetically aggregated due to a lack of nanocrystal size-dependent superparamagnetism, effectively eliminated most pathogens within an *in vitro* experiment. Achieving homogeneous distribution of IONPs throughout the biofilm was essential for eradicating the biofilm, requiring optimization of the static magnetic field (1 mm × 10 mm NdFeB magnet with a magnetic field of 1.17–1.21 T) and exposure time frames (multiple points between 0–30 min, with an optimal point at 5 min).^159^ Similar studies employing IONP assemblies combined with antibiotics reported comparable outcomes, showing enhanced antibiotic efficacy and improved biofilm removal (Supporting Information Tables S3–S5).^155,156,160^

In another study, multicore chitosan-coated IONP assemblies (80–140 nm) have also been demonstrated as polyvalent magnetic nanocarriers for bacteriophages targeting *P. aeruginosa* and *E. coli* biofilms.^161^ Upon exposure to a static magnetic field (66 mT and 52.5 kA/m), a biofilm removal efficiency of 88.7% was achieved for *P. aeruginosa*, highlighting the effective, self-replicating nature of bacteriophages and positioning them as promising antimicrobial agents for the targeted control of resistant bacterial biofilms. Another research found that applying a static magnetic field, with an NdFeB magnet (50 × 30 × 10 mm, 2000–2200 Gs) for 5 min improved the biofilm-reducing performance of multicore polydopamine-coated IONP assemblies (274 nm), compared to conditions without a magnetic field.^162^ *In vivo* studies further demonstrated that subgingival injection of these particles, combined with magnetic field application, enabled efficient delivery of magnetic carriers deep into periodontal pockets. This approach effectively reduced inflammation by facilitating antibiotic transport into the biofilm and promoting bacterial eradication.^162^

### 4.2 Spherical Multicore IONP Assemblies as Channel-Forming Delivery Systems with Magnetic Hyperthermia-Controlled Release

There are also some studies where controlled multicore IONP magnetic targeting nanosystems were developed for antibiotic delivery to bacterial biofilms, while magnetic hyperthermia was used to trigger cargo release (Supporting Information Tables S4 and S5). Multicore IONPs assemblies have emerged as a promising and versatile magnetic delivery vehicle for antibacterial agents and a remotely controlled nanotool for creating unidirectional channels deep within the biofilm matrix. Their magnetic properties enabled precise magnetic targeting and controlled antibiotic release, facilitating improved penetration and localized delivery of therapeutic agents into biofilms. For instance, Álvarez et al.^157^ developed spherical multicore IONP assemblies by anchoring several 12 nm IONPs to the surface of mesoporous silica nanoparticles capped with thermoresponsive poly­(*N*-isopropylacrylamide) (PNIPAM) (Figure 4C,E). Using a static magnetic field gradient (30 min) for targeting and AMF to generate heat, they triggered antibiotic release, reducing the *E. coli* biofilm by 4-log_10_ at a particle concentration of 200 μg/mL (Figure 4D). In a further study, 10 nm IONPs and ciprofloxacin were coentrapped into mesoporous vaterite-phase calcium carbonate particles.^163^ The constructs were magnetically targeted and accumulated, while an exposure to AMF (210 kHz; 1 kA/m and 12.6 Oe) allowed the nanosystem to rapidly release the antibiotic as the calcium carbonate transformed from porous vaterite to dense calcite. This nanosystem demonstrated biofilm reductions of 71% in *E. coli* and 85% in *S. aureus*. Another study described IONP alginate hydrogel microcapsules loaded with norfloxacin as a controlled multicore IONP magnetic delivery nanosystem. The core of the microcapsules contained low-melting hydrophobic wax, while the shell was formed by polymeric hydrogel with immobilized IONPs. The release of the antibiotic was controlled by an external radiofrequency magnetic field, which caused heating of IONPs, melting of the core wax, and antibiotic release. The efficacy of the released norfloxacin was demonstrated on *E. coli* growth, which was inhibited completely following three cycles of antibiotic release.^164^

However, magnetic targeting systems described in this section may not be sufficiently effective, as they can be guided only unidirectionally. Such IONP configurations are not optimal for multidirectional biofilm penetration, an ability that is inherent to magnetic particles with anisotropic shapes, such as magnetic micro- and nanorobots.^165^

## 5 Anisotropic Multicore IONPs and Their Hierarchical Assemblies Driven by Rotating Magnetic Fields

Despite numerous studies employing static permanent magnetic fields with associated magnetic field gradients to achieve unidirectional penetration of multicore IONPs into biofilms, major challenges remain regarding both the penetration efficiency and uniform distribution of particles within the biofilm matrix^160,166,167^ (Supporting Information Table S1). Achieving homogeneous distribution *in vivo* is particularly difficult due to the limited thickness and inaccessibility of most clinically relevant biofilms.

Unlike spherical particles, which primarily undergo unidirectional motion along magnetic field gradients, anisotropic particles can align and rotate when exposed to rotating magnetic fields at low frequencies, enabling advanced functionalities such as directional propulsion, drilling, swiping, spinning, vortexing, and therefore active penetration (Supporting Information Table S6).^168^ Particle shape anisotropy thus plays a critical role in active magnetic guidance, whereby rotating anisotropic particles convert applied magnetic torque into locomotion within viscous or structured media (e.g., mucus or biofilms), facilitating active penetration as opposed to passive diffusion. When integrated with multimagnet or advanced multielectromagnet actuation systems, shape-anisotropic particles can not only be directed toward target sites but also dynamically and temporally assembled into swarms, substantially enhancing their ability to overcome biological barriers and access otherwise inaccessible regions.^169,170^ The shape anisotropy of magnetic particles can be fully exploited by two main magnetic actuation systems available: the permanent magnetic actuation system (PMAS) and the electromagnetic actuation system (EMAS), both capable of generating rotating magnetic fields.^171^ PMAS primarily generates magnetic field gradients or torque variations and is characterized by a nonuniform magnetic field distribution, where field intensity decreases rapidly with distance.^172^ In contrast, EMAS enables more precise control of anisotropic particles’ motion but produces relatively low magnetic field intensities, which can limit the motion of anisotropic particles in complex environments.

### 5.1 Anisotropic Multicore IONP Assemblies (Magnetic Microrobots)

Multicore IONP assemblies with anisotropic shapes can be fabricated by templating IONPs onto or incorporating them into anisotropic nonmagnetic matrices,^169,175^ or can be produced through magnetic field-assisted assembly followed by fixation within a rigid matrix such as silica (Figure 6A).^154^ These anisotropic multicore IONP structures, referred to as nano- or microrobots (depending on their size), are capable of complex motion in response to rotating magnetic fields due to their high magnetic susceptibility.^176^ Advanced magnetic microrobots can perform multiple tasks in both biomedical and environmental applications (Supporting Information Table S1). Their spatial movement does not rely on toxic chemical fuels, making them especially promising for biomedical use.

In a specific application, magnetically actuated helical nanorobots with IONPs embedded in silica pillars were employed to penetrate dentinal tubules (∼4000 nanorobots per tubule with ∼1 mm depth), addressing bacterial persistence and the risk of endodontic reinfection.^176^ The nanorobots were guided by a combination of rotating and oscillating fields through the application of a triaxial Helmholtz coil generating a magnetic field (3–5 mT and 2.4–4 kA/m) rotating at 5 Hz for 20 min. It was found that heating alone by nanorobots could not eliminate the *Enterococcus faecalis* biofilm, highlighting the potential of combining physical disruption with antibiotics for better bactericidal effects.

Similar other studies employed templating or embedding strategies, such as incorporating IONPs into silica, polydopamine, or biogenic (plant-derived) matrices, to create anisotropic multicore magnetic nanostructures capable of active, magnetically driven penetration through biofilms for enhanced antibacterial and biofilm removal efficiency (Supporting Information Table S6).^174,177,178^

### 5.2 Swarms of Magnetic Microrobots as Magneto-mechanical Biofilm Destructors

Currently, one of the most advanced nanotech strategies for tackling biofilms involves the use of magnetically controlled dynamic hierarchical assemblies of nano- and microrobots, capable of reversible and adaptive swarming, rapidly assembling and disassembling in a dynamic manner into temporally and spatially defined configurations (Figure 7A,B).^169^ Swarms are well-defined, dynamic, and reversible superstructures that can perform coordinated tasks on a scale beyond the capability of individual magnetic robots (Supporting Information Table S6). In our recent study,^29^ we developed bioinspired, propelling, and dynamically assembling magnetic nanochains as a second-generation nanorobot-based strategy for enhancing antibiotic efficacy against *S. epidermidis* biofilms (Figure 6B). These ultrashort nanorobots can reversibly swarm and thus mimic the movement of swimming bacteria, which have the capability to infiltrate opponent species biofilms (Figure 7A, B, and G). These nanorobots could form transient microscopic hierarchical structures with sizes depending on the strength of the magnetic field and could be remotely actuated to form spinning swarms after exposure to low-frequency (<10 Hz), low-intensity (<20 mT) rotating magnetic fields (Figure 7G).^29^ In contrast to spherical multicore IONP assemblies creating unidirectional channels, these transient spinning swarms of nanorobots exhibit torque-driven, multidirectional movement that allowed them to penetrate and mechanically disrupt the biofilm matrix (Figure 6B). Their magnetic responsiveness, elongated shape, and negative ζ potential enabled electrostatic attachment to bacterial aggregates and facilitated physical disruption of the extracellular matrix. This magneto-mechanical agitation increased the permeability of biofilms to methicillin, resulting in a 4-log_10_ CFU reduction (99.99% reduction) in a strain considered as methicillin-resistant. This low-energy, low-cost approach demonstrates strong potential for clinical applications in treating persistent infections and for noninvasive cleaning of medical devices and infected tissue surfaces.^29^ Similarly, other studies have employed IONP-based nanostructures forming swarm-like assemblies under magnetic actuation to enhance biofilm removal (Supporting Information Table S6). Electromagnetically actuated magneto-nanozyme swarms of mesoporous IONPs achieved >6-log bacterial reduction under AMF stimulation,^179^ while reconfigurable STARS bristles performed adaptive mechanochemical cleaning of *ex vivo* human teeth.^180^ Pollen-templated urchin-like microrobots (MUCRs) achieved complete biofilm removal from biliary stents within 10 min.^181^ A similar magnetically actuated swarming strategy was demonstrated by Mayorga-Martinez et al.,^175^ who developed 400 nm magnetic nanorobots using halloysite nanotubes as structural backbones, decorated with IONPs and coated with polyethylenimine (PEI) to load ampicillin and prevent premature release. Additionally, PEI acted as a β-lactam potentiator, enabling ampicillin to kill efficiently up to 99% of bacteria from bacterial biofilms. The nanorobots exhibited multidirectional motion as individual units and as swarms, capable of transitioning between tumbling and spinning modes and switching swarm motion patterns from vortex to ribbon and back. Under magnetic actuation (10 Hz, 5 mT, and 4 kA/m, for 1 h), vortex-mode swarming enabled effective penetration and disruption of the extracellular matrix of the *S. aureus* biofilm on a titanium mesh (5 mm × 5 mm). This resulted in decrease in biofilm viability by 2 orders of magnitude, corresponding to 93% removal.^175^ In another related study, using magnetic actuation and antibiotics, by Sun et al.,^182^ a liquid-bodied magnetic robot (a dynamically cross-linked poly­(vinyl alcohol) hydrogel with embedded IONPs and loaded with levofloxacin and indolizidine) was developed, which was constructed from a dynamically cross-linked magnetic hydrogel, offering a promising strategy for removing biofilms from medical implants, achieving approximately 87% removal in stents and 84% in meshes during *in vivo* tests. This soft microrobotic platform was magnetically actuated and exhibited a tunable viscoelastic response, allowing it to conform to complex surface topographies and operate within confined anatomical spaces. Two setups actuated the antibiofilm robot: a motor-coupled 50 mm NdFeB magnet and a robotic arm for *in vitro* and *ex vivo* assays and a motor-coupled 25 mm NdFeB magnet for *in vivo* trials, with movement and rotation controlled by computer programs. Upon actuation, the robot integrated multiple mechanisms of biofilm eradication, including mechanical disruption of the extracellular matrix, chemical deactivation of bacterial cells, and collection of biofilm debris. Its performance was validated *in vitro, ex vivo* in porcine bile ducts under endoscopic and X-ray imaging, and *in vivo* in a murine model with indwelling infected implants.^182^ Another study by the same authors developed magnetic hydrogel micromachines (MHMs), soft, thermosensitive microrobots that represented a key advancement by coupling magnetic actuation with catalytic activity. These MHMs were constructed from poly­(*N*-isopropylacrylamide) (PNIPAM) hydrogels embedded with IONPs and specifically designed for biofilm removal in confined tubular environments. The embedded IONPs acted as catalytic centers, converting released hydrogen peroxide (H_2_O_2_) into bactericidal reactive oxygen species (ROS), while the thermosensitive PNIPAM matrix enabled localized, on-demand H_2_O_2_ release upon heating. These soft microrobots were magnetically actuated and operated in two distinct motion modes, planar rotation and wobbling, which enabled effective mechanical disruption of the biofilm matrix. Two magnetic actuation systems were used: (i) a Helmholtz coil for conical magnetic fields (up to 10 mT) and (ii) a motor-mounted magnet for rotating fields (up to 100 mT). Wobbling motion was achieved with 5 mT, 1–5 Hz rotation, and an 80° angle to the *y*-axis. This combined physical and chemical approach effectively eliminated biofilms in microfluidic chips, although challenges remain in complex environments like deep crevices or liquid–gas interfaces.^183^ Interestingly, Xu et al.^184^ presented a distinct approach by designing spiky hybrid nanorobots composed of IONP cores coated with gold nanocrystals embedded within a polydopamine shell, introducing both magnetic and plasmonic functionalities. Two nanorobot sizes were prepared (189 and 312 nm), both exhibiting strong photothermal properties due to the presence of gold. Upon near-infrared irradiation, these nanorobots achieved strong concentration-dependent antibacterial efficacy against *E. coli* and *S. aureus*. In addition to the photothermal effect, magneto-mechanical activity was also exploited by applying a rotating magnetic field at 5 Hz. The spiky surface morphology enhanced magneto-mechanical interactions, which were critical for biofilm disruption, resulting in over 50% biofilm removal. The rotating magnetic field further induced the *in situ* formation of temporary, chain-like swarm superstructures measuring several hundred micrometers. Larger nanorobots formed more efficient swarms or dynamic superstructures, enabling the most effective biofilm eradication through the synergistic combination of photothermal and magneto-mechanical effects.^184^

**Figure 7:**
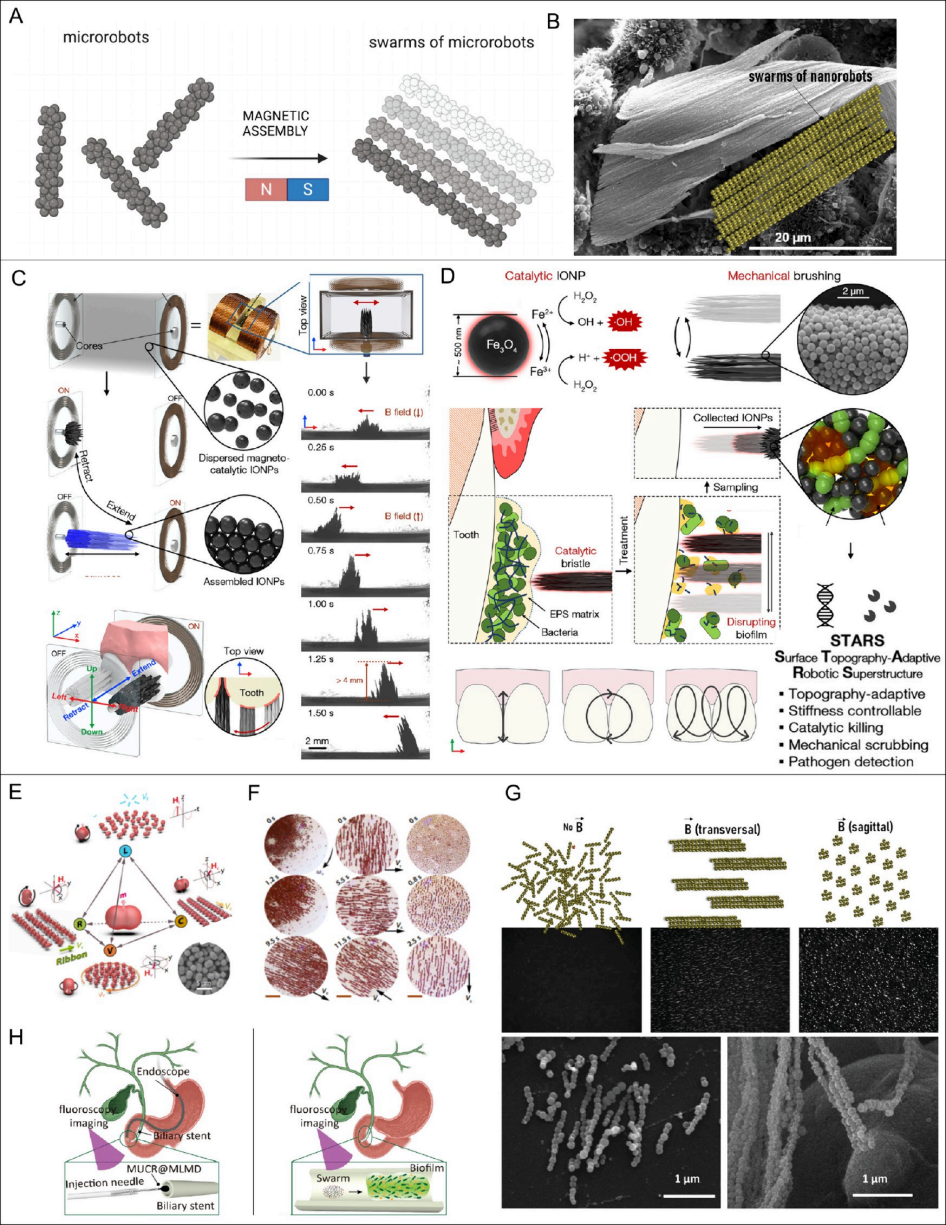
Recent strategies for biofilm treatment utilizing nano- and microrobots, which further magnetically assemble into reconfigurable swarms (A). Created with BioRender. The SEM micrographs show the assembled swarm-like structures, which were fixed with glutaraldehyde and are most likely bound together by the biological matter (B). Reproduced with permission from ref 29. Copyright 2024 Wiley-VCH GmbH. Assembly, control, and functional properties of surface topography-adaptive robotic superstructures (STARS) (C). Reprinted from ref 180. Available under CC-BY-NC-ND 4.0. Copyright 2022 ACS Nano. IONPs were assembled into a bristle-like superstructure with controllable stiffnesses. The electromagnet core guides the bristles across the target surface with a topography-adaptive property. IONPs are multifunctional with peroxidase-like activity, generating free radicals at the site of mechanical cleaning, providing both antimicrobial treatment and physical biofilm removal (D). Reprinted from ref 180. Available under CC-BY-NC-ND 4.0. Copyright 2022 ACS Nano. Representation of multimodal transformations and collective manipulation. Schematic of four programmable collective formations and transformations among them (E). Reprinted with permission from ref 169. Copyright 2019 AAAS (Science Robotics). Snapshots showing transformations to the chain from liquid, vortex, and ribbon states (F). Reprinted with permission from ref 169. Copyright 2019 AAAS (Science Robotics). Dispersed nanorobots in the absence of the magnetic field, nanorobots forming transient swarms arranged in horizontal planes, and nanorobots forming transient swarms arranged perpendicularly to the magnet and corresponding SEM images (G). Reproduced with permission from ref 29. Copyright 2024 Wiley-VCH GmbH. Schematic diagram of the eradication of biofilm adhered to biliary stents using the magnetic urchin-like capsule robots (MUCR) swarm (H). Reproduced with permission from ref 181. Copyright 2022 Wiley-VCH GmbH.

Overall, these studies demonstrate that magnetically controlled swarm-based nanorobotic systems, whether composed of IONP assemblies, antibiotic-loaded nanostructures, or multifunctional hybrid materials, represent one of the most advanced strategies for biofilm eradication. By exploiting collective motion, anisotropic design, and external magnetic actuation, these systems achieve precise navigation, active mechanical disruption, and enhanced antibacterial efficacy within complex biological environments.

## 6 Effects of IONP-Based Microrobots and Their Swarms on Mammalian Cells

Magnetic fields offer strong potential for noninvasive and localized therapeutic delivery; however, achieving effective *in vivo* targeting in human patients requires more than biocompatible magnetic carriers; it critically depends on the precise design and spatial modulation of magnetic fields, often involving multimagnet systems, to guide therapeutic agents across biological barriers over clinically relevant distances (2–5 cm).^185^ By combining physical disruption capabilities, catalytic activity, and targeted drug delivery, microrobots and their swarms represent a multifaceted therapeutic platform with complex mechanisms of action. Despite being promising, challenges related to *in vivo* applicability, long-term safety, and precise control in complex tissues remain.^186^ Beyond biofilm disruption, magnetic microrobot swarms may also interact with mammalian cells. While magnetic actuation has demonstrated clear potential for controlled modulation of cellular functions, including adhesion, proliferation, and differentiation, the hypothesis that mechanical forces alone are sufficient to induce mammalian cell destruction remains under investigation.^187^ Certain studies have shown that disk-shaped permalloy particles, when exposed to low-frequency rotating magnetic fields, can induce apoptosis and suppress tumor growth in glioma models, suggesting the potential of magnetic particle-induced mechanical ablation in both *in vitro* and *in vivo* settings.^188^ Studies suggest that magnetic actuation of intracellularly localized particles can compromise lysosomal membrane integrity, resulting in the release of proteolytic enzymes into the cytosol and subsequently triggering apoptosis.^189^

These cytotoxic effects, however, stand in contrast to recent findings demonstrating that magnetic particles can act as nanoscale actuators, stimulating mechanosensitive receptors, such as integrins and Piezo1, and activating intracellular signaling pathways including MAPK and FAK, without inducing overt cellular damage.^190^ Mazuel et al.^168^ also reported the absence of cytotoxicity, showing that iron oxide nanorods, when assembled into larger “microrods” at the cell membrane, could undergo sustained mechanical rotation under conical magnetic fields, forming hybrid biomagnetic structures with embedded membrane filaments (Figure 8).^168^ Remarkably, these macro-sized rods exhibited torsional pendulum behavior, continuing to rotate freely after the cessation of the magnetic field due to elastic energy stored in the coiled membrane structures, without compromising cell viability. In contrast to receptor-specific nanoparticle associates that may disrupt membrane integrity, these membrane-integrated microrods demonstrated an adaptive mechanical response, highlighting the resilience and elasticity of mammalian cell membranes under swarm-induced mechanical stress.^168^ Our *in vitro* studies demonstrated that magnetic nanorobots exhibit minimal cytotoxicity toward both cancerous and noncancerous cell lines, as confirmed in 2D and 3D culture models (Figure 8F).^173,191^ However, when activated by near-infrared (NIR) light, these otherwise biocompatible assemblies exhibit potent cytotoxic photothermal effects. Upon irradiation, nanochains not only induce localized hyperthermia sufficient to eradicate tumor cells *in vitro* but also cause real-time disruption of the extracellular environment by melting the collagen matrix, as observed in engineered cell sheets with a self-secreted extracellular matrix.^191^

**Figure 8:**
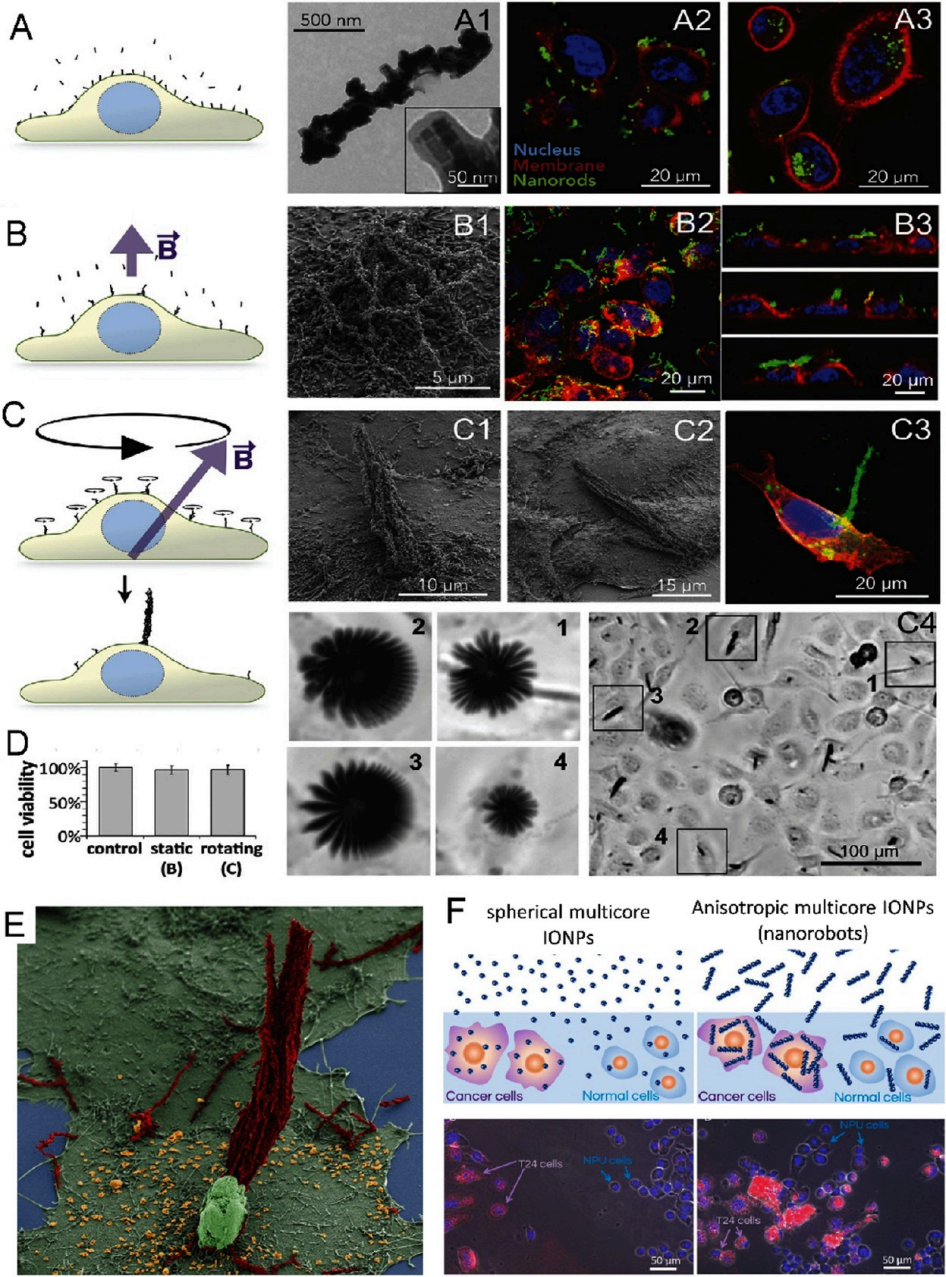
Assembly process of magnetic nanorods (nanorobots) into a microrod (microrobot) at the cell membrane with corresponding TEM, SEM, and confocal microscopy images. Nanorods dispersed in the cell culture medium interact individually with the cell membrane without a magnetic field (A). Formation of small nanorobot clusters still attached to the membrane under the application of a static vertical magnetic field (B). Rotating stimulation process: the magnetic field spins, describing a cone around its initial vertical direction. This rotating motion forces the nanorobot clusters to interact over a wider range and form a larger magnetic microrobot (C). These microrobots rotate freely in the absence of magnetic field stimulation. Cell viability measured by quantifying the metabolic activity (Alamar blue) of cells incubated with the magnetic nanorobots in the presence of the static magnetic field (condition “static,” similar to part (B)), and under the influence of the rotating magnetic field (condition “rotating,” similar to part (C)) and compared to control cells (D). Artificially colored SEM image showing microrobots in the presence of a cell (E). Figures A−E are reproduced with permission from ref 168. Copyright 2017 Wiley-VCH GmbH. Schematic illustration of the internalization process of spherical multicore IONPs and anisotropic multicore IONPs (nanorobots) in a co-culture model of normal (NPU) and cancer (T24) urothelial cells, accompanied by representative fluorescence microscopy images (F). Both nanostructure types demonstrated preferential uptake by T24 cells compared to NPU normal cells. Notably, nanorobots exhibited a higher degree of internalization into T24 cells relative to spherical multicore IONPs, while both nanostructures maintained negligible cytotoxicity under the tested conditions. Reproduced from ref 173. Available under CC BY 3.0. Copyright 2023 The Royal Society of Chemistry.

Collectively, these findings illustrate a dual paradigm: on one hand, control over magnetic robots and their swarms can be adjusted to elicit lethal mechanical damage, especially through intracellular targeting, while on the other, cells demonstrate mechanical adaptability and resilience, particularly when swarm-induced forces are distributed across membrane-integrated assemblies. Given that mammalian cells actively sense and remodel their mechanical microenvironment, establishing the threshold between mechanical stimulation and cytotoxicity will be essential for the safe and effective application of magnetic robot and swarm technologies in biomedical contexts.^192^ Moving forward, the research field must focus on improving material biocompatibility, miniaturization of magnetic actuation systems, and adaptive navigation to fully realize the clinical potential of these next-generation antibiofilm tools.

## 7 Opportunities, Challenges, and Critical Perspectives

The emergence of magnetically responsive multicore IONPs and their evolution into anisotropic nano- and microrobots represents a transformative frontier in the fight against persistent biofilms in various fields, especially in clinical infections, but also on surfaces in the food industry and other industrial or environmental systems that are susceptible to microbial contamination. These advanced magnetic superstructures, particularly those engineered to respond to rotating magnetic fields and to operate collectively in swarming formations, offer unique mechanical and chemical functionalities that go far beyond traditional antimicrobial disinfection and cleaning strategies (Supporting Information Table S1).

A major opportunity lies in the ability of these multicore and anisotropic particles to exert magneto-mechanical disruption at scales and in geometries previously inaccessible. Spherical multicore IONPs, when guided using magnetic field gradients, demonstrate channel-forming abilities within the dense extracellular matrix of biofilms. When designed with anisotropic geometries, such as rods, chains, tetrapods, or durian-like nanostructures, their response to rotating magnetic fields enables torque-driven multidirectional movement, allowing these structures to act as nanoscale drills or blades. This capability enhances biofilm penetration and promotes deeper antibiotic delivery. The reversible and adaptive swarming behavior of microrobotic systems, particularly those that assemble dynamically into temporally defined superstructures, introduces a paradigm shift in biofilm eradication. Swarms enable collective movement and increased force generation, while dynamic assembly endows the robots with spatial and surface adaptability. Structures such as surface topography-adaptive robotic superstructures (STARS), magnetic nanochains (MNC), magnetic hydrogel micromachines (MHMs), and sunflower pollen-templated microrobots (MUCRs) exemplify the range of platforms capable of navigating complex biological surfaces.^29,180,181,183^ These magnetic robots not only disrupt the physical biofilm barrier but can also carry and locally release antibiotics, ROS-generating agents, or enzymes. Nevertheless, considerable biological, toxicological, technical, and translational barriers must still be addressed, as discussed in the following sections.

### 7.1 Biological Barriers

One of the most significant of these challenges is the nature of the target itself: although magnetic nano- and microrobotic systems have demonstrated promising antibiofilm capabilities in controlled laboratory settings, their translation to clinically relevant environments remains fundamentally constrained by the intrinsic heterogeneity of biofilms. Real-world biofilms exhibit complex, multilayered physiology, including mixed-species composition, variable extracellular polymeric substance (EPS) architecture, spatially stratified metabolism, hypoxic and nutrient gradients, and the presence of host-derived components such as mucins, DNA, or immune factors. These properties critically influence mechanical stiffness, porosity, viscosity, and chemical microenvironments, all of which directly govern magnetic penetration efficiency, torque transfer, and microrobot motion within the matrix (Figure 2). However, current magnetic antibiofilm studies rarely characterize these properties or model their variability; instead, most investigations rely on simplified, single-species, laboratory-grown biofilms that lack structural and biochemical realism. As a result, there is no systematic understanding of how different magnetic modalities—magnetic hyperthermia, ROS generation, magnetic drug delivery, or magneto-mechanical actuation—perform in thick, stiff, or clinically mature biofilms, nor is it known which nanostructure types (single-core IONPs, multicore assemblies, anisotropic microrobots, or swarms) are best suited for diverse EPS compositions (protein-rich, polysaccharide-rich, or DNA-rich matrices). Addressing this major knowledge gap will require integrating detailed mechanobiological profiling of biofilms with magnetic actuation studies, establishing standardized biofilm models with tunable stiffness and composition, and systematically evaluating nanostructure performance across this spectrum. Without such efforts, predictions about *in vivo* efficacy and rational design of magnetic antibiofilm platforms remain limited, marking this area as one of the most pressing challenges for future research. As a consequence of this structural and mechanical complexity, reported antibiofilm outcomes often fall well below clinically relevant thresholds. Many studies describe statistically significant reductions, yet decreases below 99.9% represent only partial removal, which is not sufficient in practice due to the persistence of residual biofilms and may therefore be inadequate for practical biofilm control applications. Typically, a 3-log_10_ reduction (99.9%) is considered acceptable for general cleaning, while the United States Environmental Protection Agency guidelines require a ≥6-log_10_ reduction (99.9999%) in less than 10 min to claim disinfection in healthcare settings.^193,194^ Achieving such thresholds in heterogeneous, multilayered, and mechanically robust biofilms is substantially more challenging than in simplified *in vitro* models, underscoring the need for more realistic and rigorously characterized evaluation systems.

### 7.2 Toxicological Barriers

Equally important is the thorough evaluation of IONP safety in collaboration with relevant regulatory authorities. Potential risks include mechanical damage from particle aggregation, chemical toxicity, and excessive release of iron ions, all of which must be carefully characterized in preclinical and clinical studies. Uncoated or unstable nanoparticles are particularly prone to aggregation and dissolution, both of which can intensify toxic responses. Aggregated particles may physically obstruct microvasculature or other narrow biological conduits, leading to their potential toxicity. This concern is even more relevant for anisotropic particles, whose magneto-mechanical activity can exert additional forces on surrounding tissues, although these effects can be moderated by appropriate control of magnetic field strength. In addition to understanding how magneto-mechanical disruption affects surrounding healthy tissues, understanding the fate of nanoparticles in the body remains a central issue: once administered, IONPs are rapidly phagocytosed and tend to accumulate in organs of the reticuloendothelial system, such as the liver and spleen. Their metabolic degradation, routes of excretion, and half-life depend strongly on surface coatings, which influence stability, retention, and reactivity.^195^ Interactions with blood components may further complicate their safety profiles by causing hematological effects, including changes in coagulation, hemolysis, or platelet activation.^196^ Although iron oxide nanocrystals are generally well-tolerated, the coatings, surfactants, and functional moieties used for enhanced targeting or catalytic activity may introduce toxicity if not designed properly.^186^ Designing biocompatible and biodegradable surface coatings that affect the biological fate of IONPs could help mitigate the risks of long-term accumulation. For hyperthermia-based strategies, heating must remain within SAR safety limits to prevent collateral tissue damage, yet many studies report these parameters inconsistently or assess them independently of other toxicity factors. Despite these limitations, certain applications may be more feasible than others. For instance, IONPs are particularly suitable for use on the skin or mucosal surfaces, where topical or local administration avoids the complications of systemic distribution. In the oral cavity or at wound sites, their limited systemic absorption is advantageous, offering local activity with reduced systemic exposure. Similarly, hollow organs such as the stomach, intestines, bladder, gallbladder, or even cardiac chambers represent accessible sites where nanoparticles could be applied more safely and effectively than in solid organs. One intriguing possibility is the use of IONPs to disrupt and remove microbial biofilms from implants located in these hollow organs, providing a novel strategy against device-associated infections.

### 7.3 Technical Barriers

A central technical challenge lies in generating the complex magnetic fields required for controlled actuation, particularly for multicore systems and microrobotic swarms. Current platforms depend on multiaxis coils, precise field gradients, and real-time imaging, yet these setups are neither standardized nor scalable for clinical use. At the material level, inconsistent nanoparticle size and shape continue to hinder reproducibility, resulting in variable magnetic forces, uneven hyperthermia performance, and unpredictable *in vivo* behavior. Preventing aggregation under physiological conditions is equally critical, as agglomeration reduces magnetic responsiveness, increases toxicity risk, and further complicates control. Maintaining colloidal stability and responsiveness in the presence of bodily fluids, immune responses, and acidic microenvironments presents a significant hurdle. Material degradation, unintended aggregation, and insufficient penetration depth continue to restrict *in vivo* applicability. Suitable and configurable magnetic field generation is another critical bottleneck. While permanent and electromagnetic actuation systems exist, their ability to generate homogeneous and precisely controlled fields in deep tissues is constrained by equipment complexity and energy demands. A primary requirement is therefore the development of magnetic field applicators capable of delivering controlled, localized stimulation. Devices such as Helmholtz coil-based rings or tunnel applicators would need to be specifically tailored to particular body parts to ensure both efficacy and patient safety. Moreover, anisotropic particles are highly shape-dependent in their actuation behavior, meaning that small synthesis variances can result in inconsistent or suboptimal performance.

Particle anisotropy adds an additional layer of complexity, as small synthesis deviations can lead to substantial differences in actuation behavior. Compounding these issues, most current systems are tested under idealized *in vitro* or *ex vivo* conditions, with limited demonstration of efficacy in physiologically relevant *in vivo* environments. For further development, it is important to establish a threshold for antimicrobial efficacy and to set and use standardized protocols and complex biofilm models that take into account the type of biofilm treated and include a reference antimicrobial agent for comparison.^186,197^

Magnetically actuated multicore IONPs, microrobots, and microrobotic swarms represent a highly promising yet complex field with multifunctional capabilities and precision that could provide significant advantages over conventional biofilm treatments (Supporting Information Table S1). However, successful translation into clinical or environmental settings requires overcoming critical challenges related to scalable and reproducible fabrication methods, simplified magnetic actuation systems, and the establishment of robust safety profiles. Interdisciplinary collaboration among materials science, bioengineering, microbiology, and clinical medicine will be essential to fully realize the potential of these advanced magnetic superstructures. By integrating thoughtful device engineering, rigorous safety assessment, smart nanoparticle design, and careful selection of clinical indications, IONP-based technologies could progress from experimental platforms to valuable therapeutic tools for effective biofilm management and beyond. Biofilm heterogeneity adds another layer of complexity to translation. Variations in species composition, EPS density, adhesion, porosity, and mechanical stiffness mean that a strategy effective against one biofilm type may perform poorly against another.

### 7.4 Future Outlook

Building on these translational challenges, another major limitation is the lack of systematic comparative evidence across magnetic modalities. Although magnetic antibiofilm technologies have advanced rapidly, the field still lacks the systematic comparative evidence needed to determine whether increased structural or functional complexity truly enhances therapeutic efficacy. To date, each magnetic modality has been explored largely in isolation: single-core IONPs are typically evaluated for ROS generation, magnetic hyperthermia, or metal-ion-mediated antibacterial activity; spherical multicore assemblies are studied for magnetic targeting, channel formation, or antibiotic delivery; and anisotropic microrobots and swarms are tested almost exclusively under rotating magnetic fields for magneto-mechanical actuation. Although more complex nanoarchitectures could, in principle, operate across multiple modalities (e.g., combining hyperthermia, ROS generation, and drug delivery), no study has yet examined this multimodal potential in a unified manner. Conversely, simpler structures such as single-core IONPs or spherical clusters cannot participate in magneto-mechanical actuation due to insufficient torque, lack of shape anisotropy, and limited responsiveness to low-frequency rotating fields. These inherent asymmetries further complicate direct comparison between systems.

In conclusion, identifying a single optimal magnetic antibiofilm strategy is not currently feasible (Supporting Information Table S1). The absence of standardized testing platforms, the structural and physiological complexity of biofilms, and the limited exploration of multimodal or synergistic approaches all contribute to this uncertainty. Advancing the field will require systematic, controlled studies that evaluate multiple magnetic modalities within realistic, clinically relevant biofilm models and directly compare their performance. Such efforts are essential not only for determining which strategies are most effective for specific biofilm types but also for uncovering how multimodal systems may unlock higher levels of biofilm removal than any individual mechanism alone.

Looking ahead, there is a clear need for the community to design and conduct comparative multimodal studies, which remain almost entirely absent in the current literature. Most existing work evaluates only a single modality or at most combines two modalities such as hyperthermia with antibiotics or mechanical disruption with drug delivery, leaving the field largely unaware of potential synergies achievable through more complex, integrated approaches. Given the inherent heterogeneity and resilience of biofilms, it is highly plausible that engaging multiple magnetic mechanisms simultaneously could produce enhanced antibacterial effects through complementary or reinforcing modes of action. Carefully designed multimodal experiments with unified controls and quantitative end points will therefore be crucial for identifying beneficial interactions, understanding mechanistic interdependencies, and guiding the rational development of next-generation magnetic antibiofilm technologies.

## Supplementary Material



## Notes

Summary of advantages, limitations, and magnetic modalities of the investigated nanostructures; biofilm EPS composition; magnetic nanoparticle-based antimicrobial, hyperthermia, and magnetic field-actuated strategies (PDF)

## #

Department of Research and Development, Razi Vaccine and Serum Research Institute, Agricultural Research, Education and Extension Organization (AREEO), Karaj 31975, Iran

## Vocabulary

biofilm: A structured microbial community embedded in a self-secreted matrix of extracellular polymeric substances (EPS), forming a protective hydrogel that facilitates adherence to surfaces, resistance to environmental stress, and long-term microbial survival.
extracellular polymeric substances (EPS): A complex mixture of polymers, primarily polysaccharides, proteins, lipids, and extracellular DNA, secreted by microorganisms within a biofilm, providing it with structural integrity, facilitating adhesion, and creating a protective microenvironment that shields the microorganisms from physical, chemical, and biological stressors. The dynamic and adaptive nature of EPS enables water and nutrient retention, genetic exchange, immune evasion, and resistance to mechanical and antimicrobial removal, making it a key factor in biofilm persistence.
spherical multicore IONP assemblies: Spherical structures composed of numerous superparamagnetic iron oxide nanoparticles. Each nanoparticle within the assembly retains its magnetic properties, making the entire multicore structure highly responsive to magnetic fields due to the enlarged volume and hence a large magnetic moment.
anisotropic multicore IONP assemblies (microrobots): Assemblies that are composed of multiple small iron oxide nanoparticles, which are aligned under an external magnetic field, followed by fixation with an inorganic or polymeric matrix to preserve their shape. These anisotropic assemblies have a high magnetic moment while maintaining the superparamagnetic characteristics of the individual nanoparticles.
magnetic microrobot swarms: Under an external magnetic field exposure, different anisotropic magnetic particles (microrobots) further organize and form larger dynamic, adaptive, and transient groups of microrobots: swarms. These magnetically organized microrobot swarms interact with biofilms, reflecting the unique dynamics of their magnetically coordinated motion.
