# Supplementary material for: Magnetic Field-Driven Strategies for Biofilm Disruption: From Iron Oxide Nanoparticles to Adaptive Swarms of Magnetic Microrobots
Source: ACS Nano. 2026 Jan 1;20(1):34–58. doi: 10.1021/acsnano.5c14390 (PMC12810490; doi:10.1021/acsnano.5c14390)
Supplement: Supplementary file 1 [file nn5c14390_si_001.pdf]

SUPPORTING INFORMATION

Magnetic Field Driven Strategies for Biofilm Disruption: From Iron Oxide Nanoparticles to Adaptive Swarms of Magnetic Microrobots

Maja Caf[1,2], Parvaneh Esmaeilnejad-Ahranjani[1,#], Jelena Kolosnjaj-Tabi[3], Jerica Sabotič[4], Aleš Berlec[2,4], Nika Zaveršek[4], Stane Pajk[2], Abida Zahirović[4], Muriel Golzio[3], Irena Milosevic[5], Slavko Kralj[1,2]*

1. Department for Materials Synthesis, Jožef Stefan Institute, Ljubljana 1000, Slovenia

2. Faculty of Pharmacy, University of Ljubljana, Ljubljana 1000, Slovenia

3. Institut de Pharmacologie et de Biologie Structurale (IPBS), Université de Toulouse, CNRS, Université Toulouse III – Paul Sabatier (UPS), Toulouse 31400, France

4. Department of Biotechnology, Jožef Stefan Institute, Ljubljana 1000, Slovenia

5. HEPIA, University of Applied Sciences of Western Switzerland (HES-SO), Geneva 1202, Switzerland

# Present address: Department of Research and Development, Razi Vaccine and Serum Research Institute, Agricultural Research, Education and Extension Organization (AREEO), Karaj 31975, Iran

*Email: slavko.kralj@ijs.si

Table S1. Summary of key advantages, limitations, and commonly employed magnetic modalities in relation to the functional and structural complexity of the investigated nanostructures.

**Level of complexity**	**Key advantages**	**Limitations**	**Primary mechanisms of action**	**Site of action (within biofilm)**	**Practical application niches**	**Translational readiness**
**Single-core IONPs**	Easy synthesis; biocompatible; intrinsic antimicrobial activity; strong biofilm penetration; versatile delivery platforms (can carry antimicrobial coatings, metal ions, or antibiotics); enable field-triggered, on-demand hyperthermia	Weak magnetic responsiveness; limited force generation; reduced magnetically guided control	Metal-ion release; ROS generation via Fenton-like catalysis; deep diffusion into the biofilm matrix; localized hyperthermia; targeted delivery	Outer and intermediate layers, primarily for drug/vector delivery	Drug carriers, antibiofilm coatings, catheter irrigation, imaging-guided delivery	High (clinically approved formulations exist (e.g., ferumoxytol); relatively well-characterized safety; simplest to translate; one already in clinical trials – NCT06110494 [1])
**Multicore IONP clusters**	Higher magnetic moment; enhanced force generation; strong magnetic responsiveness; effective carriers for antimicrobial agents (e.g., antibiotics, enzymes, metal ions); and excellent performance in magnetic hyperthermia, enabling field-triggered, on-demand thermal disruption.	Prone to aggregation with potential loss of superparamagnetism; challenging size/shape uniformity; more complex fabrication	Magnetically driven mechanical disruption and channel formation; strong force/torque generation; localized hyperthermia; targeted delivery	Intermediate layer (microcolonies & EPS-rich zones) with partial access to basal layer	Surface-associated biofilms, topical treatments	Moderate (limited by toxicity concerns and lack of standardized safety evaluation)
**Magnetic microrobots**	Strong mechanical disruption; deep biofilm	Technically complex; demanding	High-intensity mechanical shear	Biofilm–liquid interface and	Device-associated	Low (currently no viable clinical

	penetration; programmable motion	fabrication; high system complexity; limiting practical testing and demonstration; toxicity concerns	and drilling, combined with targeted cargo delivery	upper/intermediate layers, possible access to basal layer via deep channel digging and navigation	biofilms, endoscope channels, stents, tubing	actuation systems; complex compositions, potential biodegradability issues)
**Microrobot swarms**	Strong mechanical disruption; deep biofilm penetration; programmable motion; collective swarm enhancement, high adaptability	Technically complex; demanding fabrication; high system complexity; limiting practical testing and demonstration; niche applications; toxicity concerns	High-intensity mechanical shear and drilling, combined with targeted cargo delivery	Interface and outer layers, with collective erosion of intermediate layer; partial detachment of basal structures	Large-surface biofouling, endoscopic cleaning, environmental biofilms, pipeline systems	Very low (currently no viable clinical actuation systems; high regulatory hurdles; potential safety, control, and biodegradability concerns)

Table S2. A table summarizing biofilm EPS components.

EPS Component	Intra-molecular Bonds	Molecular Charge	Possible Inter-molecular Bonds	Function in Biofilms
**Polysaccharides**	Glycosidic bonds (α- or β-linkages between monosaccharides)	Usually neutral or negatively charged (due to uronic acids, sulfated groups), positive charge also possible	Hydrogen bonds, ionic interactions (with cations or proteins), van der Waals forces	Scaffold formation<br>Structural integrity and stability<br>Cell-cell binding<br>Adhesion<br>Cohesion<br>Water retention<br>Ion binding<br>Immune evasion<br>Protection from antimicrobials

**Proteins (including enzymes, lectins, adhesins, amyloids and other fibrillar proteins)**	Peptide bonds, disulfide bridges, hydrophobic interactions, hydrogen bonds	Variable (depends on amino acid composition; often amphoteric)	Hydrogen bonds, ionic bonds, hydrophobic interactions with other proteins or polysaccharides	Structural integrity and stability<br>Mechanical strength<br>Adhesion to surfaces and cells<br>Cohesion<br>Matrix remodeling<br>Signaling<br>Nutrient acquisition<br>Metabolism<br>Immune evasion<br>Detachment and dispersion
**Extracellular DNA (eDNA) and RNA**	Phosphodiester bonds	Negatively charged (phosphate backbone)	Ionic interactions (mainly with cations and positively charged proteins), hydrogen bonds	Structural integrity and stability<br>Initial adhesion<br>Source of organic carbon, nitrogen and phosphate<br>Horizontal gene transfer<br>DNA damage repair<br>Ion sequestration<br>Protection from antimicrobial agents (e.g. cationic antimicrobial peptides)
**Lipids and glycolipids**	Ester bonds within fatty acids and glycerol, glycosidic bonds	Mostly hydrophobic; some amphiphilic<br>Neutral or negatively charged (phosphate or acidic sugars)	Hydrophobic, van der Waals interactions, hydrogen bonds	Hydrophobic barrier formation<br>Adhesion to surfaces<br>Signaling<br>Structural stability<br>Immune evasion<br>Cohesion
**Ions and other (small) molecules**	Ionic and coordination bonds	Variable	Electrostatic interactions, bridging between charged macromolecules	Adaptability<br>Cohesion<br>Structural stability<br>Signaling<br>pH buffering<br>Metal sequestration<br>Nutrients<br>Microenvironment creation

Table S3. An overview of magnetic nanoparticle-based antimicrobial studies.

Type of particles/size/shape	Functionalization/ conjugation	Targeted bacteria	Results	Mode of action	Ref
SPIONs/ 8 nm/ cubic	/	*S. epidermidis (ATCC 35984)*	SPIONs reduced *S. epidermidis* viability and biofilm formation at doses as low as 10 µg/mL, with higher doses (100 µg/mL–2 mg/mL) showing stronger effects over 12–48 hours.	Bactericidal effect through ROS generation and electrostatic binding of nanoparticles to bacterial membranes, disrupting membrane integrity and vital cell functions.	[2]
SPIONs/ 6-9 nm/ spherical	/	*E. coli (ATCC 25922), P. aeruginosa (PAO1), Serratia marcescens (ATCC 13880), L. monocytogenes*	Sub-inhibitory concentrations (1/16–1/2 × MIC) of SPIONs inhibited biofilm formation by *P. aeruginosa* (16–82%), *E. coli* (28–77%), *L. monocytogenes* (22–88%), and *S. marcescens* (19–75%) in a dose-dependent manner.	ROS generation through Fenton reaction leading to oxidative damage of bacterial cells.	[3]
IONPs/10-120 nm*/ spherical	/	*S. aureus (MTCC 1144), Bacillus licheniformis (MTCC 7425), Bacillus brevis (MTCC 7404), S. aureus (Lab isolate), S. epidermidis (MTCC 3615), B. subtilis (MTCC 7164)*	IONPs exhibited broad-spectrum moderate antibacterial activity, with inhibition zones up to 22 mm, particularly effective against *Bacillus* spp. and *Staphylococcus* spp., while showing no activity against *P. aeruginosa* and *S. flexneri*.	ROS generation through Fenton reaction leading to oxidative damage of bacterial cells.	[4]
IONPs/ 213 ± 26 nm	/	*S. mutans UA159*	IONPs (0.5 mg/mL) combined with 1% H_2O_2 achieved >99.9% bacterial killing (>5-log reduction (log red)) within 5 min, over 5000× more effective than H_2O_2 alone.	Peroxidase-like activation of H_2O_2 under acidic pH, generating ROS that degrade EPS and kill bacteria; trace Fe release also reduces enamel demineralization.	[5]

Bare, amine, carboxyl coated SPIONs/ 10.1 ± 0.6 nm, 11.4 ± 0.4 nm and 12.1 ± 0.5 nm/ spherical	Bare, amine, carboxyl	*S. mutans (NCTC 10449)*	SPIONs surface charge affected antibacterial efficacy against *S. mutans* biofilms, with positively charged SPIONs most toxic, followed by bare and negatively charged ones.	Enhanced electrostatic interaction enables positively charged SPIONs to penetrate negatively charged biofilms, causing greater bacterial killing, while surface functionalization and protein adsorption further influence toxicity.	6
IONPs/ 10-20 nm/ spherical	Bare, chitosan	*B. subtilis (MTCC 736), E. coli (MTCC 443)*	Uncoated IONPs (n-IONPs) caused ~30 % (0.16 log red) reduction in bacterial viability at 50 µM, chitosan-coated IONPs (p-IONPs) achieved ~70 % (0.52 log) reduction for both *Bacillus subtilis* and *Escherichia coli*, and LIVE/DEAD assays confirmed ~90 % non-viable cells after 50 µM p-IONPs treatment.	p-IONPs kill bacteria via ROS-mediated oxidative stress and electrostatic membrane disruption.	7
IONPs/ 6-15 nm/ spherical	Citric acid, amine, aminosilane, arginine, oleic acid	*S. aureus, E. coli*	The oleic acid–coated IONPs showed the highest antibacterial and antibiofilm efficiency — reducing bacterial growth by 83 % (S. aureus) and 79 % (E. coli) at 400 µg/mL and inhibiting biofilm formation by up to 94 % (S. aureus) and 96 % (E. coli); they also destroyed preformed biofilms by 94 % and 93 %, respectively.	Surface interaction–driven membrane disruption and oxidative stress.	8
IONPs/ 50-100 nm/ spherical	OA	*S. aureus, Pseudomonas aeruginosa*	OA-IONPs showed stronger antibiofilm activity against Gram-positive *S. aureus* than Gram-negative *P. aeruginosa*, significantly reducing *S. aureus* biofilm growth and metabolism.	OA-IONPs generate ROS, inhibit the FabI enzyme essential for fatty acid synthesis, and reduce bacterial adhesion and biofilm formation, particularly in Gram-positive bacteria.	9
SPIONs/ >20 nm/ quasi-spherical	Bare, OA	*S. aureus (MTCC 1144)*	Bare IONPs generated fewer ROS but exhibited the highest bacterial inhibition,	Antibacterial effect via Fenton reaction–mediated ROS generation	10

			likely due to mechanical damage from their crystalline structure.	and membrane rupture; OA coating reduces ROS and mechanical damage by limiting iron release.	
Silver NPs, IONPs/ 35, 48 nm/ spherical	Rhamnolipid (RL)	*S. aureus*, *P. aeruginosa*	RL-IONPs removed up to 91 % of biofilms, while RL-Ag NPs achieved 88 %, representing a 3–4-fold improvement over uncoated or RL-only treatments.	ROS-induced oxidative stress from IONPs (and Ag) nanoparticles, enhanced by rhamnolipids that disrupt the biofilm matrix and promote nanoparticle penetration and bacterial damage.	[11]
IONPs/ 9 ± 2 nm/ spherical	Gold, aminosilane	*E. coli (PCM 2268), S. aureus (PCM 2267), P. aeruginosa (PCM 2270), C. albicans (PCM 2566)*	Bare IONPs inhibited *E. coli* (39%, 0.22 log red) and *P. aeruginosa* (97%, 1.52 log red) but not *S. aureus*. Gold-coated IONPs fully suppressed *P. aeruginosa* and reduced *C. albicans* by (90%, 1 log red). Aminosilane-coated *IONPs* inhibited *E. coli* (75%, 0.6 log red), *S. aureus* (26%, 0.13 log red), *P. aeruginosa* (99%, 2 log red), and *C. albicans* (~84%, 0.80 log red).	Membrane disruption via electrostatic interactions and cell wall damage; IONPs aminosilane functionalized$_2$ also internalized into cells, enhancing killing.	[12]
IONPs/32.5 ± 14.2, 14.7 ± 3, 11.4 ± 1.8, 15.6 ± 3.6, 32.2 ± 9.6 nm/ spherical	Dextran	*S. mutans UA159 (ATCC 700610)*	Among dextran formulations (1.5–40 kDa), 10 kDa offered optimal catalytic activity, biofilm uptake, and antibacterial properties, reducing bacterial viability by 6-log red and significantly lowering biomass.	Dextran-coated iron oxide nanozymes catalytically activate H_2O_2 to produce ROS that selectively destroy acidic bacterial biofilms by bacterial killing and EPS degradation, while sparing healthy tissues.	[13]
Magnesium ferrite nanoparticles ($MgFe_2O_4$)/ 25-35 nm/ semi-spherical	/	*E. coli (ATCC 25922), S. aureus (ATCC 25923)*	Treatment with MgFe2O4 NPs significantly inhibited biofilm formation, with *E. coli* showing 89% inhibition and *S. aureus* 78.5% inhibition at a concentration of 10 µg/mL.	ROS generation and membrane disruption lead to oxidative stress, DNA damage, and ion transport inhibition.	[14]

Poly(vinyl alcohol) coated IONPs/ 9 nm ± 4 nm/ self-assembly into chain-like structures	/	*S. aureus (ATCC 25923)*	IONPs significantly reduced *S. aureus* viability at highest dose 3 mg/mL after 4, 12, and 24 hours.	ROS generation through Fenton reaction leading to oxidative damage of bacterial cells.	15
Fe–Co oxide nanocrystals/ 100 nm length, 20 nm width/ spheroidal nanorods	Graphene oxide, poly-tannic acid	*E. coli (MG1655, ATCC 25922), S. aureus (ATCC 25923)*	The NPs achieved a ~5 log (99.999%) reduction in *E. coli* and *S. aureus* biomass and up to ~85% biofilm removal (*S. aureus* 85.2%, *E. coli* 83.5%).	MTex-500, a mesoporous iron oxide nanoparticle, kills bacteria primarily by catalyzing H_2O_2 to generate ROS. These ROS cause oxidative damage to bacterial membranes, leading to cell death.	16
SPIONs/ 10.07 ± 1.54 nm/ spherical	Dimercaptosuccinic acid (DMSA), Ag	*methicillin-resistant S. aureus (MRSA, Mu50, ATCC 700699)*	At 1 mg/mL, silver-conjugated SPIONs reduced planktonic MRSA growth to ~58% and biofilm mass by ~30%; under a magnetic field, planktonic growth dropped further to ~22% and biofilm mass to ~47%, demonstrating enhanced antibacterial efficacy.	Silver-conjugated SPIONs kill bacteria by releasing Ag^+ ions that generate ROS and disrupt membranes, while their magnetic core enhances biofilm penetration and intracellular ion uptake under an external magnetic field.	17
IONPs and Ag NPs embedded into gum arabic/polyvinyl alcohol/polycaprolactone (GA/PVA/PCL) nanofiber composites/ N.A./ nanofibers	/	*S. aureus (IBRC-M 10917), P. aeruginosa (ATCC 27853)*	Nanocomposites with 15% IONPs inhibited S. aureus and P. aeruginosa biofilms by ~14–15% (0.07 log red), while exposure to external magnetic field eradicated >90% (1 log red) of the biofilm.	The nanocomposites acted via ROS generation, membrane disruption, and magnetically enhanced nanoparticle and ion penetration into biofilms.	18
IONPs immobilizing polyphenol oxidase via glutaraldehyde crosslinking /IONPs: 50–100 nm/ cross-linked enzyme aggregates	3-aminopropyltriethoxysilane	*Escherichia coli (MTCC 405), Staphylococcus aureus (MTCC 3160), Klebsiella pnuemoniae (MTCC 432), MRSA (ATCC 43300), Candida albicans (ATCC 90028)*	70–75% reduction) biofilm inhibition (E. coli, S. aureus, K. pneumoniae, MRSA); 64% for C. albicans; retained ~70% efficiency after 5 reuse cycles.	Inhibits microbial adhesion proteins and reduces EPS, preventing cell attachment and biofilm formation.	19

IONPs used to form magnetic combi cross-linked enzyme aggregates loaded with (cellulase, pectinase, xylanase/ IONPs: 50–100 nm/ cross-linked enzyme aggregates	3-aminopropyltriethoxysilane	*E. coli (MTCC 405), S. aureus (MTCC 3160)*	75–78% biofilm inhibition against *E. coli* and *S. aureus*, showing higher efficiency than the free enzyme (58–63%) and maintaining activity for up to four reuse cycles.	The m-combi-CLEA disrupted biofilm formation by enzymatically degrading polysaccharides in the EPS matrix, weakening cell adhesion and leading to biofilm disintegration.	[20]

Values marked with an * indicate that the nanoparticle size was determined using Dynamic Light Scattering rather than Transmission Electron Microscopy or Scanning Electron Microscopy.

Table S4. A table summarizing magnetic hyperthermia studies for biofilm removal.

Type of particles/size/shape	Functionalization/conjugation	Conditions of AMF	Targeted bacteria	Combination with antibiotics	Results	Ref
SPIONs - BioMag® Superparamagnetic Iron Oxide/ N.A./spherical	Bare	3 kA/m, 37.7 Oe, 492 kHz, 8min	*P. aeruginosa (PA01)*	/	4-log red at 62.3 °C.	[21]
IONPs 10 ± 2 nm /spherical	Poly-acrylic acid	100 Oe, 873 kHz, 8 min	*P. fluorescens ATCC 27663*	/	≥3-log red and planktonic eradication at 55 °C.	[22]
IONPs/ 160 nm* / /mesoporous spherical	Polyethylene glycol	2.5 kW, 210 kHz	*E. coli (CICC 10 389), S. aureus (CICC 21600)*	/	73.9% (0.58-log red) *E. coli* and 70.2% (0.53-log red) *S. aureus* killed at 43°C.	[23]
IONPs/ 70–80 nm/ spherical	Bare and polyethylene imine	/	*E. coli (ATCC 25922) S. aureus (ATCC 25923), P. aeruginosa (MCCC 1A00099)*	/	≤5-log red of planktonic and sessile bacteria and 87% (0.89-log red) biofilm reduction at 45°C.	[24]
nanomag-D-SPIO/ 100 nm/ spherical	/	18, 24, and 30 kA/m, 2.1 MHz, 6 min	*S. aureus (ATCC 6538)*	Ciprofloxacin	2-log red of *S. aureus* biofilm with ciprofloxacin combined with AMF.	[25]

Magnetic nanoparticles/ N.A./ spherical	/	60 kA/m, 375 kHz, 10 min	*S. aureus (ATCC 6538 strain), P. aeruginosa (PAO1, ATCC BAA-47)*	Ciprofloxacin	2-log red of *S. aureus* and 3-log red of *P. aeruginosa* in diabetic wounds with AMF combined with ciprofloxacin.	26
IONPs /12 nm / spherical	Poly((oligo(ethylene glycol) methyl ether acrylate)-*block*-poly(monoacryloxy ethyl phosphate)	196 kHz, 20 min	*P. aeruginosa (PAO1)*	Gentamicin	2-log red of *P. aeruginosa* in planktonic and biofilm phases with nanocomposite and gentamicin under AMF (3.2–4.1× more effective than gentamicin alone).	27
$CoFe_2O_4$ and $MnFe_2O_4$ /70.88 ± 25.05 nm*/ spherical	Nitrous acid	1.35 kA/m	*S. aureus (ATCC 43300), E. coli (ATCC 25922),*	/	Generated heat (50 °C/5 min) and released nitric oxide (80 μmol L^{-1}/10 min), achieving ~2.5-log red of biofilm bacteria and near-complete eradication of sessile bacteria on implants.	28
IONPs/ N.A./ N.A.	Streptavidin	18, 31, and 40 kA/m, 2.1 MHz, 3 min	*S. aureus (SH1000)*	/	3-log (~99.9%) red of in-vitro *S. aureus* biofilm and ~80% in-vivo wound reduction with improved healing at 43°C.	29
Cu/C/Fe_3O_4 /1.5 µm in length, 300 nm in diameterFe_3O_4 150 nm, Cu 60 nm/ nanorods	Carboxyl	0.14–0.25 W/cm², 2.45 GHz, 10–17 minute	*S. aureus (ATCC 25923), MRSA (CICC 16465)*	/	4-log red of *S. aureus* and ~2.4-log red of MRSA in vitro via microwave-irradiated nanoparticles; *in vivo*, microwave treatment with magnetic targeting treated osteomyelitis and prevented bacterial spread.	30

Mesoporous silica nanoparticles (MSN)decorated with SPIONs (12± 2 nm)/ 255 nm*/ spherical	Amino and methacrylate groups / poly(ethylene glycol) / poly(N-isopropylacrylamide) thermo-responsive polymer / SPIONS / levofloxacin	202 kHz, 30 mT, 30 min intervals, until reaching a final time of 240 min	*E. coli (ATCC 25922)*	Levofloxacin	Antibiotic-free nanosystem under AMF (~50 °C) achieved ~2-log *E. coli* biofilm reduction; levofloxacin loaded nanosystem achieved ~4-log red (200 µg mL^{-1}, 16 h).	31
Mesoporous vaterite $CaCO_3$ microparticles co-entrapping SPIONs and 5 wt% ciprofloxacin / $CaCO_3$ (~1.3 µm), SPIONs (10 nm)/ spherical	Ciprofloxacin	210 kHz, 1 kA/m, 1 min	*E. coli (ATCC 263-116), S. aureus (209P)*	Ciprofloxacin	~0.54-log red of *E. coli* biomass and ~0.82-log red of *S. aureus* biofilms under AMF (65 °C); ciprofloxacin encapsulation enhanced activity by 72% vs free drug.	32
Core–shell microcapsules with a wax core (loaded with norfloxacin) and an alginate shell embedded with iron-oxide nanoparticles / microcapsules 300–500 µm, IONPs: 5.6 ± 0.9 nm/ spherical	Norfloxacin	900 kHz, 3 min at 100 % + 1 min at 45 %	*E. coli (EC43)*	Norfloxacin	Above 35 °C, radiofrequency-triggered release produced clear inhibition zones on agar; 3 RF pulses released ~ 4 % of drug and completely killed *E. coli* in suspension.	33
Self-assembly SPIONs/ 241 ± 68 nm / microswarms	/	AMF (513 kHz at 1001.1 A/m, 8 kW, and 28.2 A, 15 min	S. aureus (MRSA, ATCC 43300)	/	>6-log kill and 96% MRSA biofilm mass removal with MNPs + H_2O_2 under EMS + AMF (~45 °C, 15 min); *in vivo*, ~99.99% (4-log red) reduction with enhanced wound healing.	34

*Values marked with an ** indicate that the nanoparticle size was determined using Dynamic Light Scattering rather than Transmission Electron Microscopy or Scanning Electron Microscopy.

Table S5. A table summarizing studies on magnetic nanoparticles as antibacterial delivery vehicles (channels forming agents).

Type of particles/size/shape	Delivered Agents or Compounds	Targeted bacteria	Results	Mode of action	Ref
IONP/ 12 ± 2 nm/ spherical	Chlorhexidine	*methicillin-sensitive S. aureus* (MSSA), *E. faecalis, Escherichia coli* (ESBL), *P. aeruginosa* Xen 5 (MDR), *Candida* sp.	CHX-functionalized MNPs enhanced antimicrobial efficacy, lowering MIC/MBC or MFC/MBIC up to 32× and inhibiting biofilm formation (>50%) in *S. aureus*, *E. coli*, and *C. albicans*.	Incubation with antibiotic-functionalized nanoparticles.	[35]
SPION/13–15 nm/ quasi-spherical	Dextran coated, loaded with curcumin	*E. coli ATCC 25922, S. aureus ATCC 25923, C. albicans ATCC 10231*	Curcumin loading was ~15–60% with ~27–37% released by day 7, yielding 5–8 mm inhibition zones, reduced MICs and up to ~4× biofilm suppression, while remaining biocompatible.	Incubation with functionalized nanoparticles.	[36]
IONP/ 5–15 nm/ spherical	O-aryl-carbamoyl-oxymino-fluorene derivatives	*E. coli ATCC 25922, S. aureus ATCC 1026, C. albicans ATCC 10231*	The nanoparticles showed enhanced antibacterial and antifungal effects compared to the bare compounds, with MIC values 2–8 times lower.	Incubation with functionalized nanoparticles.	[37]
IONP/ 15 nm ± 2 nm / spherical	Amphotericin B (17%), nystatin (32%)	*C. albicans, C. glabrata, C. tropicalis*	Polyene-functionalized MNPs lower drug toxicity and boost antifungal efficacy. Iron oxide nanoparticles (IONPs) inactivate catalase (Cat-1), overcoming resistance and enhancing host immune system.	Incubation with antibiotic-functionalized nanoparticles.	[38]
Mesoporous IONPs/ <100 nm / spherical	APTES-functionalized, loaded with ciprofloxacin	*S. aureus (ATCC-23235)*	Ciprofloxacin loaded into the particles at about 95% for IONPs and about 88% for APTES-IONPs (with 150–300 µg/mL feed). In S. aureus biofilms, the loaded IONPs (50 mg/mL carrier, ~132–143 µg/mL cipro) almost completely broke up the	Incubation with antibiotic-functionalized nanoparticles.	[39]

			biofilm and worked better than free ciprofloxacin at 300 µg/mL, while unloaded particles did little.		
SPION/ 41 nm*/ N.A.	Poly (acrylic acid) coated, Bovine serum albumin conjugated, mannoside-tagged, loaded with ciprofloxacin	*E. coli (Isolate no 230 and 294)*	Ciprofloxacin-loaded, mannoside-tagged SPIONs lowered the killing dose for resistant E. coli from 128→32 µg/mL (~97% kill of planktonic cells). In biofilms, 32 µg/mL cut bacteria by 86.9% in a thick (dense, high-mass) biofilm and 98.5% in a thin (less-dense) biofilm—reducing Minimum Biofilm Eradication Concentration (MBEC) to 32 µg/mL (16× and 8× lower, respectively).	Incubation with antibiotic-functionalized nanoparticles.	[40]
IONPs/ 20.51 and 36.23 nm/ spherical	Gentamicin	*S. epidermidis, Proteus mirabilis, Acinetobacter baumannii*	In the disc-diffusion test, gentamicin alone produced inhibition zones of 17.5 mm for *P. mirabilis*, 17.6 mm for *A. baumannii*, and 17.3 mm for *S. epidermidis*; when gentamicin was loaded onto PEG-coated iron oxide nanoparticles, the zones increased to 25.6, 26.3, and 23.6 mm, respectively.	Incubation with functionalized nanoparticles.	[41]
IONP/ 60 nm/ spherical	Gentamicin	*Enterobacter cloacae BS 1037, S. aureus ATCC 12600, Klebsiella pneumonia-1, Acinetobacter baumannii-1, P.aeruginosa PA01, E.*	Under a 5-min magnetic field, gentamicin-loaded MNPs achieved about threefold higher biofilm-killing efficiency compared to gentamicin alone or non-magnetized controls.	Magnetic-field–assisted penetration and homogeneous distribution of gentamicin-functionalized magnetic nanoparticles through biofilms, enabling deep antibacterial killing via localized gentamicin action.	[42]

		faecalis 1396, E. coli ATCC 25922			
SPION/ 30 nm/ spherical	Gentamicin	*S. aureus (ATCC 25922), E. coli (ATCC 25923)*	Nanoparticles showed 21.8% loading capacity and 43.1% efficiency, with pH-responsive gentamicin release (~37% at pH 5.5, ~9% at pH 7.4). They achieved 92–100% antibacterial activity (highest in acidic media) and near-complete biofilm eradication under a magnetic field.	Magnetic-field–assisted penetration and pH-responsive release of gentamicin from IONPs, enabling deep biofilm penetration and enhanced antibacterial killing through localized gentamicin action.	[43]
SPIONs encapsulating polymersomes (IOPs)/ 83 ± 6 nm*/ spherical	Methicillin	S. epidermis strain RP62a (ATCC 35984)	IOPs achieved complete eradication of methicillin-resistant *Staphylococcus epidermidis* biofilms when treated under a magnetic field using the formulation containing 40 µg/mL SPIONs and 20 µg/mL methicillin.	Magnetic-field–guided positioning of iron oxide–encapsulating polymersomes beneath biofilms using aligned neodymium magnets, enabling controlled nanoparticle penetration and uniform antibiotic delivery throughout the biofilm structure.	[44]
Polycaprolactone microspheres with IONPs (maghemite/hematite)/ 55-530 nm* (maghemite), 190-1,110 nm* (hematite)/ spherical	Ciprofloxacin	*S. aureus (ATCC 2913)*	In this study, applying an alternating magnetic field increased ciprofloxacin release from the polycaprolactone–iron oxide microspheres (maghemite: ~51%→~78% at day 1; hematite: ~36%→~39%), yielding stronger suppression of S. aureus—especially with maghemite-loaded carriers.	AMF-triggered release of ciprofloxacin from polycaprolactone–iron-oxide microspheres (maghemite/hematite) increase local drug levels at the biofilm.	[45]
SPIONs embedded in mesoporous	Ciprofloxacin	*E. coli (ATCC 263-116), S. aureus (209P)*	The composite loaded ~5 wt% ciprofloxacin and released ~97% by passive vaterite→calcite conversion in	Magnet-guided, AMF-triggered: a magnet pre-localizes cipro-loaded	[32]

calcium carbonate (vaterite) micro-spheres/ 1.3 µm/ spherical			water (~5 h), or a ~49% burst followed by release to ~96% under an alternating magnetic field (210 kHz, 1 kA/m); in biofilms it reduced biomass by ~71% (E. coli) and ~85% (S. aureus) versus ~53% and ~46% for free ciprofloxacin (i.e., up to ~72% better), while magnetite alone cut biomass by ~18% and ~34%, respectively.	composite at the biofilm; the alternating field heats magnetite, driving vaterite→calcite conversion and rapid fragmentation that (i) releases cipro in situ, (ii) disrupts the matrix, and (iii) adds magnetite's ROS/cation and alkalinity effects for synergistic killing.	
Mesoporous silica nanoparticles (MSNs), coated with a thermo-responsive PNIPAM polymer, decorated with SPIONs/ ~255 nm*/ spherical	Levofloxacin	*E. coli (ATCC 25922)*	The antibiotic-free nanosystem reduced *E. coli* biofilm by 2 log under AMF, while the levofloxacin -loaded nanosystem achieved a stronger 4 log red.	Magnet-guided, AMF-triggered delivery: a neodymium magnet pre-localizes levofloxacin -loaded, PNIPAM-gated mesoporous silica nanoparticles decorated with SPIONs onto the E. coli biofilm; then the alternating magnetic field heats the surface SPIONs, causing PNIPAM to shrink and open the pores for burst levofloxacin release while mild local hyperthermia disrupts the biofilm matrix.	[31]
SPIONs embedded in an alginate shell/ N.A./ spherical	Dextran coated, norfloxacin loaded	*E. coli K12 (EC43)*	A radiofrequency magnetic field was used to heat the IONPs, melting the wax core and releasing the antibiotic. This system allowed for multiple controlled release cycles, successfully eliminating E. coli growth after three cycles.	Radiofrequency-triggered release: an alternating magnetic field heats the dextran-stabilized iron-oxide NPs in the alginate shell, melting the paraffin core so micronized norfloxacin migrates to the shell–water	[33]

				interface and dissolves; when the field stops the core re-solidifies, pausing release—allowing on-demand dosing that kills *E. coli*.	
Ureidopyrimidinone (UPy) coated IONPs / 135 ± 55 nm/ short fibers	(3-aminopropyl)triethoxysilane and poly(glutaraldehyde) coated and functionalized with UPy conjugated with lasioglossin III	*E. coli (BL21, DE3)*	UPy-coated IONPs showed ~99% binding efficiency for lasioglossin (up to ~0.68 g peptide per g NP), yielding an MIC ~1.77 µM against *E. coli* (lower than for free peptide)	Incubation with functionalized nanoparticles.	[46]

*Values marked with an ** indicate that the nanoparticle size was determined using Dynamic Light Scattering rather than Transmission Electron Microscopy or Scanning Electron Microscopy.

Table S6. A table summarizing studies on magnetic-field actuated systems (anisotropic assemblies, microrobots, and swarms).

Type of particles/size/shape	Type of actuation	Functionalization/conjugation	Targeted bacteria	Results	Ref
SPIONs, FluidMAG-CMX / 150 nm/ spherical	Static field (4.44 kG, 6 h) and switched static field (top/bottom alternation every 30 min, 6 h) generated by molybdenum magnets; oscillating field (474 kHz, 20 V, 4.3 A, 30 min) produced by a magneTherm system; combined setup used 1 h static followed by 30 min oscillating exposure.	/	*P. aeruginosa* PAO1	Combination ciprofloxacin and SPIONs exposed to switched magnetic field resulted in the greatest reduction in both metabolic activity and biomass.	[47]
IONPs/ 278 ± 61 nm*/ spherical	Static NdFeB magnet (1.17–1.21 T), cycled below, above, and around wells × 3 to form channels.	/	*S. aureus (ATCC 12600 and 5298)*	IONPs (500 µg mL^{-1}) magnetically moved for 9 min carved ~1.3–1.4 µm channels in *S. aureus* biofilms, giving a 4–6× greater CFU reduction with gentamicin versus gentamicin alone.	[48]
Carboxymethyl chitosan (CMCS)-coated IONPs/ 268.9 ± 5.3 nm*/ spherical	Static permanent magnet (355 ± 30 G) placed below the sample for 5 min to drive CMCS-coated IONPs into the biofilm for enhanced penetration and disruption.	Carboxymethyl chitosan	*S. aureus* (ATCC 25923), *E. coli DH5α*	CMCS-coated IONPs (2.0 mg/mL) under a magnetic field reduced *S. aureus* and *E. coli* biofilms by 84% (0.80-log red) and 95% (1.3-log red) after 48 h, completely eradicated planktonic cells within 10 h and 5 h, and showed no cytotoxicity to mammalian cells.	[49]

IONPs and IONPs polydopamine coated/ bare IONPs: 211 nm*/ spherical	NdFeB magnet (10 mm in diameter × 1 mm, 1.2 T) used for 5–10 min—under wells *in vitro* and moved circularly above infection sites *in vivo.*	/	*S. aureus* Xen36 (5298)	Unmodified IONPs under magnetic field enhanced gentamicin killing of *S. aureus* biofilms (from ~48% (0.28 log red) to ~80% (0.7 log red)) and, to a lesser extent, non-EPS-producing *S. aureus* biofilms; PDA-modified MIONPs showed weaker effects due to aggregation.	[50]
IONPs coated with polydopamine and loaded with minocycline (IONPs-PDA-Mino)/ 273.7 nm* / spherical	*In vitro*: NdFeB magnet (50×30×10 mm, 2000–2200 Gs) applied for 5 min to drive NPs into biofilms.<br>*In vivo*: NdFeB magnet (100×50×20 mm, 3200–3500 Gs) applied for 15 min to target NPs into periodontal pockets.	Polydopamine and loaded with minocycline	*S. sanguinis (ATCC 10556), P. gingivalis (ATCC 33277), F. nucleatum (ATCC 10953)*	IONPs-PDA-Mino showed potent antibacterial and anti-biofilm activity; under a magnetic field, they achieved the greatest bacterial killing, deep biofilm penetration, and inflammation reduction (IL-1β, IL-6, TNF-α) *in vivo*, with excellent biocompatibility.	[51]
Carboxyl-functionalized IONPs with gentamicin/ 60 nm/ spherical	Static NdFeB magnet (10 mm in diameter and 1 mm in thickness,1.17–1.21 T) was placed beneath the biofilm sample for different exposure times (0, 1, 2, 5, 10, and 30 minutes) to drive and distribute the NPs through the biofilm.	Carboxyl functionalized, loaded with gentamicin	*Enterobacter cloacae* BS 1037, *S. aureus* (ATCC 12600), *K. pneumonia*-1, *Acinetobacter baumannii*-1, *P. aeruginosa* PA01, *E. faecalis* 1396, and *E. coli* (ATCC 25922)	Under 5-minute magnetic-field exposure, NPs achieved nearly complete eradication of *S. aureus* biofilms, killing bacteria uniformly throughout the entire 53 µm thickness.	[42]

SPIONs coated with chitosan (CS) or PEG, loaded with gentamicin (G) /3 nm + 5 nm coating/ spherical	N.A.	Chitosan and PEG, loaded with gentamicin	*S. aureus (ATCC 25922), E. coli (ATCC 25923)*	Magnet-assisted IONP-PEG-G NPs achieved almost complete biofilm removal and bacterial killing, while without the magnet, only partial disruption occurred.	43
Carboxymethyl chitosan (CMCS)–grafted IONPs and CMCS-IONPs grafted with Ag / 250 nm/ spherical	Static external magnetic field was applied below the sample wells to attract and concentrate the IONPs-CMCS-Ag nanoparticles toward the biofilm surface, enhancing their contact with the *S. aureus* biofilm.	Carboxymethyl chitosan and grafted with Ag	*E. coli (BL21), S. aureus (ATCC6538 P)*	For planktonic *E. coli*, 100% killing was achieved within 30 minutes at a nanoparticle concentration of 2 mg/mL, compared to ~75% reduction with CMCS-IONPs without Ag.	52
Nanocomposite of iron oxide and silver/ 5 and 20 nm/ spherical	N.A.	Starch	*Methicillin-sensitive S. aureus DSM20231*	Nanocomposites achieved >90% biofilm eradication under a magnetic field, with ~65% remaining without the field; in a collagen gel model, magnetic exposure enabled full penetration and nearly complete bacterial eradication.	53
Silver ring–coated SPIONs and silver ring on gold-coated SPIONs/18–25 nm/ spherical	N.A.	/	*S. aureus (ATCC 19636), S. epidermidis (ATCC 35984)*	SPION–Ag and SPION–Au–Ag achieved >80% biofilm reduction under magnetic field.	54
SPIONs conjugated with antibacterial silver salts/ SPIONS:10 ± 1.5 nm, SPIONs-Ag: 176.5 ± 27* nm/ spherical	Static NdFeB magnets placed beneath each well during biofilm treatment.	Ag	*S. aureus Mu50 (ATCC 700699)*	Silver-conjugated SPIONs reduced MRSA biofilm by 30% (~0.2 log red) compared to 47% (~0.3 log red) under magnetic field and planktonic growth by 58%(~0.4 log red) compared to 78% (~0.7 log red) under magnetic field.	17

Silver ring–coated SPIONs and silver ring on gold-coated SPIONs/18–25 nm/ spherical	N.A.	/	*S. aureus (ATCC 19636), S. epidermidis (ATCC 35984)*	SPION–Ag and SPION–Au–Ag achieved >80% biofilm reduction (~0.7 log red) under magnetic field.	[54]
SPIONs conjugated with antibacterial silver salts/ SPIONS:10 ± 1.5 nm, SPIONs-Ag: 176.5 ± 27 nm*/ spherical	Static NdFeB magnets placed beneath each well during biofilm treatment.	Ag	*S. aureus Mu50 (ATCC 700699)*	Silver-conjugated SPIONs reduced MRSA biofilm by 30% (~0.2 log red) compared to 47% (~0.3 log red) under magnetic field and planktonic growth by 58% (~0.4 log red) compared to 78% (~0.7 log red) under magnetic field.	[17]
IONPs/ 22 ± 2 nm (spheres), 8 ± 2 nm (cube), 13 ± 3 nm (tetrapods)	110 mT neodymium permanent magnets rotating at 21 mHz beneath the biofilms, at 0.5 cm below the sample surface.	Cetyltrimethylammonium bromide (CTAB)	*MRSA*	Active delivery of CTAB-loaded magnetic nanoparticles (5 mg/mL, 10 min) effectively inactivated MRSA biofilms, with spheres achieving a 7.3-log red and cubes/ tetrapods reaching 14.19-log red.	[55]
Silica-coated IONPs/ ~8 nm, ~11 nm, ~70 nm/ 8 and 11 nm spherical, 70 nm polyhedral	Rotating neodymium permanent magnets (110 mT, ~0.02 Hz) positioned beneath biofilm samples for 15 min, or AC magnetic field (191 kHz, 15.8 kA m^{-1}, 15 min) generated by a 3-turn induction coil.	/	MRSA (ATCC 33592)	Under direct contact, AC, and DC rotating magnetic fields, 8 nm IONPs achieved 3.40, 3.49, and 4.00 log red; 11 nm IONPs achieved 3.54, 3.62, and 4.71 log red; and 70 nm IONPs achieved 0.98, 1.12, and 2.69 log red, respectively.	[56]
Iron-oxide–encapsulating polymersomes / polymersomes: 83 nm,	Static NdFeB (~0.5 T) placed below the sample to magnetically drive SPION-loaded nanocarriers into the	Methicillin	*S. epidermidis (ATCC 35984)*	Complete biofilm eradication under static field, ≤60% reduction without field, non-toxic to mammalian cells.	[44]

SPIONs 5 ± 2.5 nm/ spherical	biofilm for uniform drug distribution.				
SPIONs coated with poly(acrylic acid) (PAA), loaded with gentamicin, decorated with Ag nanoparticles/ 200–250 nm/ spherical cluster-like aggregates	Static NdFeB magnet (0.2 T) placed below wells to guide MNPs@Ag@HA into biofilms for enhanced penetration and eradication.	Hyaluronic acid	*S. aureus (ATCC 25922), E. coli (ATCC 25923)*	SPIONs-Ag-HA showed limited efficacy, with 94% (0.04 log red) and 71% (0.5 log red) bacterial survival 500 and 1000 µg/mL, respectively. With magnetic field, bacterial survival dropped to 5% (1.3 log red) and 0.01% (4 log red) at 500 and 1000 µg/mL, respectively.	[57]
IONPs/100.70 ± 17.33 nm/nanoclusters	Static NdFeB magnet (1.17–1.21 T) applied below the sample for 5 min to drive GOx-modified IONPs nanoparticles into the biofilm, enhancing penetration and ROS-mediated disruption.	Glucose Oxidase (GOx)	*E. faecalis* (ATCC 29212) and *C. albicans (ATCC10231)*	E. faecalis biofilm thickness decreased from 30 to 5 µm and C. albicans from 14 to 2 µm using GOx-modified particles with a magnetic field.	[58]
IONP, IONP-SiO_2–NH_2, chitosan (CS)-IONP/ 50–100 nm, 100–220 nm, 80-140 nm/ nanoclusters	Magnetic field (~660 G) applied below the sample to direct CS-IONP-PEL1 through the biofilm, enhancing penetration, mechanical disruption, and bacteriophage infection.	Polyvalent phage PEL1	*E. coli C3000 (ATCC 15597), P. aeruginosa PA01 (ATCC 15692)*	CS-IONP-PEL1 achieved 88.7% biofilm reduction after 6 h under magnetic field.	[59]
Core–shell NPs (~70 nm) with silver cores and magnetite shells/ 25–35 nm and 20 nm/ flower-structured nanocomposites	N.A.	/	*E. coli (CMCC(B)44102), P. aeruginosa (ATCC 27853)*	Under 30 min exposure to magnetic field nanocomposite (100 µg/mL) reduced *E. coli* and *P. aeruginosa* biofilms by 88% and 97%, respectively.	[60]

Nanoparticles with Fe_3O_4 core, gold nanospike layer, and PDA coating/189 and 312 nm/ shape of durian fruit	A rotating magnetic field (300 rpm, 180 mT) was applied to induce nanoparticle motion and enhance bacterial disruption and biofilm penetration.	/	*E. coli CMCC44102, S. aureus* CMCC(B)26003	Under combined NIR light (2 W cm^{-2}, 808 nm) and rotating magnetic field (300 rpm, 180 mT), Fe_3O_4-Au-PDA nanodurians (200 µg/mL) achieved nearly 100% killing of planktonic *E. coli* and *S. aureus* and for biofilm, smaller NPs removed ~54.6% (*E. coli*) and 45.0% (*S. aureus*), while larger achieved ~64.7% (*E. coli*) and 61.0% (*S. aureus*)—equivalent to roughly 0.35–0.45 log red.	61
Porous tea-bud microparticles decorated with IONPs coated with chitosan/ ~50–100 µm/ pseudo-spherical	Magnetic actuation was performed using a neodymium bar magnet (0.01 T) manually aligned or rotated to generate linear and spinning fields that propelled nanoparticles across *P. aeruginosa* and *S. aureus* biofilms grown on six-well plates.	Coated with chitosan, loaded with ciprofloxacin	*P. aeruginosa* MTCC 2488, *S. aureus* MTCC 96	Biofilm removal efficiency: 77% for *S. aureus* and 74.2% for *P. aeruginosa* under linear motion, increasing to 76.1% and 79.2%, respectively, under spinning motion.	62
IONPs- polydopamine hollow rods (IONPs-PDA HR)/ 393 nm in length, 121 nm in width/ nanorods	Rotating neodymium permanent magnets operating at 2000 rpm beneath the biofilm samples.	Fosfomycin	*S. aureus*	Exposure IONPs-PDA HR to a rotational magnetic field (20 min) reduced preformed S. aureus biofilm biomass by 65.3%, while NIR laser irradiation (10 min) achieved a 72.5% reduction through photothermal effects.	63

poly(N-isopropylacrylamide) hydrogel embedded with IONPs and NdFeB microparticles (MHMs)/ Cylindrical shape	Three magnetic setups were employed: 1. a three-axis Helmholtz coil (≤10 mT, 1–5 Hz) for wobbling motion in tubes, a rotating NdFeB ball magnet (~100 mT) for planar rotation on flat surfaces, and AMF coil for magnetothermal heating to trigger H_2O_2 release.	/	*E. coli, Bacillus cereus*	MHMs combined with H_2O_2 achieved synergistic mechanical and catalytic activity, resulting in extensive biofilm disruption and near-complete clearance of biomass compared to control and static treatments.	[64]
IONPS attached onto halloysite nanotubes/ ~400 nm long/ tubular shape	A transversal rotating magnetic field (5 mT, 0.3–10 Hz) was generated by a six-coil electromagnetic setup for microrobot actuation and biofilm disruption, with biofilm samples on glass slides or titanium mesh immersed in the microrobot suspension and placed inside the six-coil setup.	Polyethylenimine coated and ampicillin loaded	S. aureus	Dynamic magnetic actuation of nanoparticles reduced *S. aureus* biofilm viability by four orders of magnitude and achieved ~93% biofilm removal on titanium meshes, outperforming static or antibiotic-free controls.	[65]
IONPs embedded into silica pillars with a thin silver layer /250 nm, 1-3 µm in length/ helical spiral-shaped nanobots	A triaxial Helmholtz coil generated rotating (~50 G, 5 Hz) and oscillating (~50 G, 40 Hz) magnetic fields, with the biofilm grown inside dentinal tubules of human tooth samples placed at the center of the coil.	APTES-functionalized and conjugated with fluorophores (6-aminofluorescein) or upconversi	E. Faecalis (ATCC29212)	The magnetic nanobots achieved ~2000 µm penetration depth and near-complete killing of *E. faecalis* biofilms via localized magnetic hyperthermia, demonstrating much higher efficiency than existing root canal disinfection techniques.	[66]

		on nanoparticles			
IONPs (CARs) (also embedded in agar hydrogel3D molded CARs)/213 ± 26.5 nm/ spherical (rod-like structures under magnetic field)	Magnetic actuation was achieved using a 175 mT permanent magnet with micromanipulator control for biohybrid CARs and a 3.4 mT rotating field (2–4 Hz) from nested Helmholtz coils for 3D molded CARs.	/	*S. mutans UA159*	Nanoparticles in an H_2O_2–enzyme solution formed rod-like structures under a magnetic field, achieved 100% biofilm removal with no viable cells or biomass detected after treatment.	[67]
IONPs/ 464.9 ± 36.08 nm/spherical (rod-like structures under magnetic field)	Programmable dual-electromagnet setup (13–96 mT, 8 mm gap), generating lateral/circular motions (6–48 mm s^{-1}) to assemble and actuate IONPs bristles for biofilm removal on vertically positioned tooth surfaces.	/	*S. mutans UA159, C. albicans SC5314*	The treatment achieved over 90% (1 log red) biofilm removal and complete bacterial killing, with no viable cells detected.	[68]
Composite of IONPs deposited on urchin-like sunflower pollen capsules (MUCRs) containing encapsulated Galinstan–Fe magnetic liquid-metal nanodroplets/ ~10 µm/Urchin-like spherical capsules with spiky	Three-axis Helmholtz coil (0–30 mT, 0–15 Hz) and spherical NdFeB magnet on robotic arm (0–600 mT, 0–15 Hz) generating rotating magnetic fields for swarm formation and actuation.	/	*Enterococcus faecalis, Escherichia coli*	The microswarms achieved >99% bacterial killing and ~5–6 µm biofilm thickness reduction after 30–60 min of magnetic actuation, resulting in complete biofilm removal from patient-derived biliary stents in *ex vivo* and *in vivo* tests.	[69]

surface (swarm-like under magnetic field)					
SPIONs /241 ± 68 nm/ spherical mesoporous nanoclusters, microswarm under electromagnetic actuation system	Rotating magnetic field (EMS, 10 mT, 4–6 Hz, 45 min) for mechanical disruption; AMF (513 kHz, 1001.1 A/m, 15 min) for magnetothermal enhancement.	/	MRSA (ATCC,43300)	SPIONs/H_2O_2/EMS/AMF achieved ~96% biofilm removal and >6-log bacterial reduction *in vitro*; >99.99% bacterial elimination and full wound healing *in vivo*.	[34]
IONPs and Ag NPs embedded into gum arabic/polyvinyl alcohol/polycaprolactone (GA/PVA/PCL) nanofiber composites/ N.A./ nanofibers	N.A.	/	*S. aureus (IBRC-M 10917), P. aeruginosa (ATCC 27853)*	Nanocomposites with 15% IONPs inhibited S. aureus and P. aeruginosa biofilms by ~14–15% (0.07 log red), while exposure to external magnetic field eradicated >90% (1 log red) of the biofilm.	[18]

Silica-coated neodymium–iron–boron (NdFeB) magnetic microparticles embedded in a polyvinyl alcohol–boronic hydrogel/ 0.5–20 mm	Robotic arm–mounted rotating NdFeB spherical magnet (25–50 mm Ø), generating up to ~28 mT fields with ~1 T/m gradient; enables translational, rotational, and combined actuation for *in vitro*, *ex vivo*, and *in vivo* biofilm removal.	Levofloxacin, indolicidin	*E. coli (ATCC 25922), MRSA (ATCC 43300)*	Magnetic hydrogel robot achieved 84.25% biofilm biomass reduction on hernia mesh *ex vivo*, ~87% nonviable cells and 81.82% (0.74 log red) reduction on metallic biliary stents *ex vivo*, and 93.45% (1.18 log red) reduction on infected stents *in vivo*.	[70]

*Values marked with an ** indicate that the nanoparticle size was determined using Dynamic Light Scattering rather than Transmission Electron Microscopy or Scanning Electron Microscopy.

REFERENCES

(1) *Clinicaltrials.gov*. https://clinicaltrials.gov/study/NCT06110494?utm_source=chatgpt.com&tab=history (accessed 2025-11-26).

(2) Taylor, E. N.; Webster, T. J. The Use of Superparamagnetic Nanoparticles for Prosthetic Biofilm Prevention. *Int. J. Nanomedicine* **2009**, *4* (2), 145–152.

(3) Al-Shabib, N. A.; Husain, F. M.; Ahmed, F.; Khan, R. A.; Khan, M. S.; Ansari, F. A.; Alam, M. Z.; Ahmed, M. A.; Khan, M. S.; Baig, M. H.; Khan, J. M.; Shahzad, S. A.; Arshad, M.; Alyousef, A.; Ahmad, I. Low Temperature Synthesis of Superparamagnetic Iron Oxide (Fe3O4) Nanoparticles and Their ROS Mediated Inhibition of Biofilm Formed by Food-Associated Bacteria. *Front. Microbiol.* **2018**, *9*, 2567.

(4) Behera, S. S.; Patra, J. K.; Pramanik, K.; Panda, N.; Thatoi, H. Characterization and Evaluation of Antibacterial Activities of Chemically Synthesized Iron Oxide Nanoparticles. *World J. Nano Sci. Eng.* **2012**, *02* (04), 196–200.

(5) Gao, L.; Liu, Y.; Kim, D.; Li, Y.; Hwang, G.; Naha, P. C.; Cormode, D. P.; Koo, H. Nanocatalysts Promote Streptococcus Mutans Biofilm Matrix Degradation and Enhance Bacterial Killing to Suppress Dental Caries in Vivo. *Biomaterials* **2016**, *101*, 272–284.

(6) Javanbakht, T.; Laurent, S.; Stanicki, D.; Wilkinson, K. J. Relating the Surface Properties of Superparamagnetic Iron Oxide Nanoparticles (SPIONs) to Their Bactericidal Effect towards a Biofilm of Streptococcus Mutans. *PLoS One* **2016**, *11* (4), e0154445.

(7) Arakha, M.; Pal, S.; Samantarrai, D.; Panigrahi, T. K.; Mallick, B. C.; Pramanik, K.; Mallick, B.; Jha, S. Antimicrobial Activity of Iron Oxide Nanoparticle upon Modulation of Nanoparticle-Bacteria Interface. *Sci. Rep.* **2015**, *5* (1), 14813.

(8) Shebl, R. I.; Farouk, F.; Azzazy, H. M. E.-S. Effect of Surface Charge and Hydrophobicity Modulation on the Antibacterial and Antibiofilm Potential of Magnetic Iron Nanoparticles. *J. Nanomater.* **2017**, *2017*, 1–15.

(9) Velusamy, P.; Chia-Hung, S.; Shritama, A.; Kumar, G. V.; Jeyanthi, V.; Pandian, K. Synthesis of Oleic Acid Coated Iron Oxide Nanoparticles and Its Role in Anti-Biofilm Activity against Clinical Isolates of Bacterial Pathogens. *J. Taiwan Inst. Chem. Eng.* **2016**, *59*, 450–456.

(10) Aadinath, W.; Muthuvijayan, V. Influence of Oleic Acid Coating on the Magnetic Susceptibility and Fenton Reaction-Mediated ROS Generation by the Iron Oxide Nanoparticles. *Nano Ex.* **2024**, *5* (1), 015017.

(11) Khalid, H. F.; Tehseen, B.; Sarwar, Y.; Hussain, S. Z.; Khan, W. S.; Raza, Z. A.; Bajwa, S. Z.; Kanaras, A. G.; Hussain, I.; Rehman, A. Biosurfactant Coated Silver and Iron Oxide Nanoparticles with Enhanced Anti-Biofilm and Anti-Adhesive Properties. *J. Hazard. Mater.* **2019**, *364*, 441–448.

(12) Niemirowicz, K.; Swiecicka, I.; Wilczewska, A. Z.; Markiewicz, K. H.; Surel, U.; Kułakowska, A.; Namiot, Z.; Szynaka, B.; Bucki, R.; Car, H. Growth Arrest and Rapid Capture of Select Pathogens Following Magnetic Nanoparticle Treatment. *Colloids Surf. B Biointerfaces* **2015**, *131*, 29–38.

(13) Naha, P. C.; Liu, Y.; Hwang, G.; Huang, Y.; Gubara, S.; Jonnakuti, V.; Simon-Soro, A.; Kim, D.; Gao, L.; Koo, H.; Cormode, D. P. Dextran-Coated Iron Oxide Nanoparticles as Biomimetic Catalysts for Localized and PH-Activated Biofilm Disruption. *ACS Nano* **2019**, *13* (5), 4960–4971.

(14) El-Khawaga, A. M.; Ayman, M.; Hafez, O.; Shalaby, R. E. Photocatalytic, Antimicrobial and Antibiofilm Activities of MgFe2O4 Magnetic Nanoparticles. *Sci. Rep.* **2024**, *14* (1), 12877.

(15) Tran, N.; Mir, A.; Mallik, D.; Sinha, A.; Nayar, S.; Webster, T. J. Bactericidal Effect of Iron Oxide Nanoparticles on Staphylococcus Aureus. *Int. J. Nanomedicine* **2010**, *5*, 277–283.

(16) Kumari, N.; Kumar, S.; Karmacharya, M.; Dubbu, S.; Kwon, T.; Singh, V.; Chae, K. H.; Kumar, A.; Cho, Y.-K.; Lee, I. S. Surface-Textured Mixed-Metal-Oxide Nanocrystals as Efficient Catalysts for ROS Production and Biofilm Eradication. *Nano Lett.* **2021**, *21* (1), 279–287.
(17) Durmus, N. G.; Webster, T. J. Eradicating Antibiotic-Resistant Biofilms with Silver-Conjugated Superparamagnetic Iron Oxide Nanoparticles. *Adv. Healthc. Mater.* **2013**, *2* (1), 165–171.
(18) Eghbalifam, N.; Shojaosadati, S. A.; Hashemi-Najafabadi, S. Role of Bioactive Magnetic Nanoparticles in the Prevention of Wound Pathogenic Biofilm Formation Using Smart Nanocomposites. *J. Nanobiotechnology* **2023**, *21* (1), 161.
(19) Noori, R.; Bano, N.; Ahmad, S.; Mirza, K.; Mazumder, J. A.; Perwez, M.; Raza, K.; Manzoor, N.; Sardar, M. Microbial Biofilm Inhibition Using Magnetic Cross-Linked Polyphenol Oxidase Aggregates. *ACS Appl. Bio Mater.* **2024**, *7* (5), 3164–3178.
(20) Perwez, M.; Mazumder, J. A.; Noori, R.; Sardar, M. Magnetic Combi CLEA for Inhibition of Bacterial Biofilm: A Green Approach. *Int. J. Biol. Macromol.* **2021**, *186*, 780–787.
(21) Park, H.; Park, H.-J.; Kim, J. A.; Lee, S. H.; Kim, J. H.; Yoon, J.; Park, T. H. Inactivation of Pseudomonas Aeruginosa PA01 Biofilms by Hyperthermia Using Superparamagnetic Nanoparticles. *J. Microbiol. Methods* **2011**, *84* (1), 41–45.
(22) Rodrigues, D.; Bañobre-López, M.; Espiña, B.; Rivas, J.; Azeredo, J. Effect of Magnetic Hyperthermia on the Structure of Biofilm and Cellular Viability of a Food Spoilage Bacterium. *Biofouling* **2013**, *29* (10), 1225–1232.
(23) Li, W.; Wei, W.; Wu, X.; Zhao, Y.; Dai, H. The Antibacterial and Antibiofilm Activities of Mesoporous Hollow Fe3O4 Nanoparticles in an Alternating Magnetic Field. *Biomater. Sci.* **2020**, *8* (16), 4492–4507.
(24) Liu, W.; Pei, W.; Moradi, M.; Zhao, D.; Li, Z.; Zhang, M.; Xu, D.; Wang, F. Polyethyleneimine Functionalized Mesoporous Magnetic Nanoparticles with Enhanced Antibacterial and Antibiofilm Activity in an Alternating Magnetic Field. *ACS Appl. Mater. Interfaces* **2022**, *14* (16), 18794–18805.
(25) Alumutairi, L.; Yu, B.; Filka, M.; Nayfach, J.; Kim, M.-H. Mild Magnetic Nanoparticle Hyperthermia Enhances the Susceptibility of Staphylococcus Aureus Biofilm to Antibiotics. *Int. J. Hyperthermia* **2020**, *37* (1), 66–75.
(26) Almutairi, L. A.; Yu, B.; Dyne, E.; Ojaym, A. A.; Kim, M.-H. Mild Magnetic Hyperthermia Is Synergistic with an Antibiotic Treatment against Dual Species Biofilms Consisting of S. Aureus and P. Aeruginosa by Enhancing Metabolic Activity. *Int. J. Hyperthermia* **2023**, *40* (1), 2226845.
(27) Nguyen, T.-K.; Duong, H. T. T.; Selvanayagam, R.; Boyer, C.; Barraud, N. Iron Oxide Nanoparticle-Mediated Hyperthermia Stimulates Dispersal in Bacterial Biofilms and Enhances Antibiotic Efficacy. *Sci. Rep.* **2015**, *5* (1), 18385.
(28) Wang, J.; Wang, L.; Pan, J.; Zhao, J.; Tang, J.; Jiang, D.; Hu, P.; Jia, W.; Shi, J. Magneto-Based Synergetic Therapy for Implant-Associated Infections via Biofilm Disruption and Innate Immunity Regulation. *Adv. Sci. (Weinh.)* **2021**, *8* (6), 2004010.
(29) Kim, M.-H.; Yamayoshi, I.; Mathew, S.; Lin, H.; Nayfach, J.; Simon, S. I. Magnetic Nanoparticle Targeted Hyperthermia of Cutaneous Staphylococcus Aureus Infection. *Ann. Biomed. Eng.* **2013**, *41* (3), 598–609.
(30) Ren, J.; Qiao, Y.; Jin, L.; Mao, C.; Wang, C.; Wu, S.; Zheng, Y.; Li, Z.; Cui, Z.; Jiang, H.; Zhu, S.; Liu, X. A Smart Bacteria-Capture-Killing Vector for Effectively Treating Osteomyelitis through Synergy under Microwave Therapy. *Small* **2024**, *20* (15), e2307406.
(31) Álvarez, E.; Estévez, M.; Gallo-Cordova, A.; González, B.; Castillo, R. R.; Morales, M. D. P.; Colilla, M.; Izquierdo-Barba, I.; Vallet-Regí, M. Superparamagnetic Iron Oxide Nanoparticles Decorated Mesoporous Silica Nanosystem for Combined Antibiofilm Therapy. *Pharmaceutics* **2022**, *14* (1), 163.

(32) Rumyantceva, V.; Rumyantceva, V.; Andreeva, Y.; Tsvetikova, S.; Radaev, A.; Vishnevskaya, M.; Vinogradov, V.; Drozdov, A. S.; Koshel, E. Magnetically Controlled Carbonate Nanocomposite with Ciprofloxacin for Biofilm Eradication. *Int. J. Mol. Sci.* **2021**, *22* (12), 6187.
(33) Navrátil, O.; Lizoňová, D.; Slonková, K.; Mašková, L.; Zadražil, A.; Sedmidubský, D.; Štěpánek, F. Antibiotic Depot System with Radiofrequency Controlled Drug Release. *Colloids Surf. B Biointerfaces* **2022**, *217* (112618), 112618.
(34) Ma, X.; Wang, L.; Wang, P.; Liu, Z.; Hao, J.; Wu, J.; Chu, G.; Huang, M.; Mair, L. O.; Huang, C.; Xu, T.; Ying, T.; Tang, X.; Chen, Y.; Cai, X.; Zheng, Y. An Electromagnetically Actuated Magneto-Nanozyme Mediated Synergistic Therapy for Destruction and Eradication of Biofilm. *Chem. Eng. J.* **2022**, *431* (133971), 133971.
(35) Tokajuk, G.; Niemirowicz, K.; Deptuła, P.; Piktel, E.; Cieśluk, M.; Wilczewska, A.; Dąbrowski, J.; Bucki, R. Use of Magnetic Nanoparticles as a Drug Delivery System to Improve Chlorhexidine Antimicrobial Activity. *Int. J. Nanomedicine* **2017**, *12*, 7833–7846.
(36) Chircov, C.; Ștefan, R.-E.; Dolete, G.; Andrei, A.; Holban, A. M.; Oprea, O.-C.; Vasile, B. S.; Neacșu, I. A.; Tihăuan, B. Dextran-Coated Iron Oxide Nanoparticles Loaded with Curcumin for Antimicrobial Therapies. *Pharmaceutics* **2022**, *14* (5), 1057.
(37) Vlad, I. M.; Nuță, D. C.; Ancuceanu, R. V.; Caproiou, M. T.; Dumitrascu, F.; Marinas, I. C.; Chifiriuc, M. C.; Măruţescu, L. G.; Zarafu, I.; Papacocea, I. R.; Vasile, B. Ștefan; Nicoară, A. I.; Ilie, C.-I.; Ficai, A.; Limban, C. New O-Aryl-Carbamoyl-Oxymino-Fluorene Derivatives with MI-Crobicidal and Antibiofilm Activity Enhanced by Combination with Iron Oxide Nanoparticles. *Molecules* **2021**, *26* (10), 3002.
(38) Niemirowicz, K.; Durnaś, B.; Tokajuk, G.; Głuszek, K.; Wilczewska, A. Z.; Misztalewska, I.; Mystkowska, J.; Michalak, G.; Sodo, A.; Wątek, M.; Kiziewicz, B.; Góźdź, S.; Głuszek, S.; Bucki, R. Magnetic Nanoparticles as a Drug Delivery System That Enhance Fungicidal Activity of Polyene Antibiotics. *Nanomedicine* **2016**, *12* (8), 2395–2404.
(39) Lage, W. C.; Sachs, D.; Nunes Ribeiro, T. A.; Tebaldi, M. L.; de Moura, Y. dos R. S.; Domingues, S. C.; Ferreira Soares, D. C. Mesoporous Iron Oxide Nanoparticles Loaded with Ciprofloxacin as a Potential Biocompatible Antibacterial System. *Microporous Mesoporous Mater.* **2021**, *321* (111127), 111127.
(40) Atac, N.; Onbasli, K.; Koc, I.; Yagci Acar, H.; Can, F. Fimbria Targeting Superparamagnetic Iron Oxide Nanoparticles Enhance the Antimicrobial and Antibiofilm Activity of Ciprofloxacin against Quinolone-Resistant E. Coli. *Microb. Biotechnol.* **2023**, *16* (11), 2072–2081.
(41) Abdulsada, F. M.; Hussein, N. N.; Sulaiman, G. M.; Al Ali, A.; Alhujaily, M. Evaluation of the Antibacterial Properties of Iron Oxide, Polyethylene Glycol, and Gentamicin Conjugated Nanoparticles against Some Multidrug-Resistant Bacteria. *J. Funct. Biomater.* **2022**, *13* (3), 138.
(42) Quan, K.; Zhang, Z.; Ren, Y.; Busscher, H. J.; van der Mei, H. C.; Peterson, B. W. Homogeneous Distribution of Magnetic, Antimicrobial-Carrying Nanoparticles through an Infectious Biofilm Enhances Biofilm-Killing Efficacy. *ACS Biomater. Sci. Eng.* **2020**, *6* (1), 205–212.
(43) Wang, X.; Deng, A.; Cao, W.; Li, Q.; Wang, L.; Zhou, J.; Hu, B.; Xing, X. Synthesis of Chitosan/Poly (Ethylene Glycol)-Modified Magnetic Nanoparticles for Antibiotic Delivery and Their Enhanced Anti-Biofilm Activity in the Presence of Magnetic Field. *J. Mater. Sci.* **2018**, *53* (9), 6433–6449.
(44) Geilich, B. M.; Gelfat, I.; Sridhar, S.; van de Ven, A. L.; Webster, T. J. Superparamagnetic Iron Oxide-Encapsulating Polymersome Nanocarriers for Biofilm Eradication. *Biomaterials* **2017**, *119*, 78–85.
(45) Sirivisoot, S.; Harrison, B. S. Magnetically Stimulated Ciprofloxacin Release from Polymeric Microspheres Entrapping Iron Oxide Nanoparticles. *Int. J. Nanomedicine* **2015**, *10*, 4447–4458.

(46) Turrina, C.; Cookman, J.; Bellan, R.; Song, J.; Paar, M.; Dankers, P. Y. W.; Berensmeier, S.; Schwaminger, S. P. Iron Oxide Nanoparticles with Supramolecular Ureido-Pyrimidinone Coating for Antimicrobial Peptide Delivery. *Int. J. Mol. Sci.* **2023**, *24* (19). https://doi.org/10.3390/ijms241914649.
(47) Bandara, H. M. H. N.; Nguyen, D.; Mogarala, S.; Osiñski, M.; Smyth, H. D. C. Magnetic Fields Suppress Pseudomonas Aeruginosa Biofilms and Enhance Ciprofloxacin Activity. *Biofouling* **2015**, *31* (5), 443–457.
(48) Quan, K.; Zhang, Z.; Chen, H.; Ren, X.; Ren, Y.; Peterson, B. W.; van der Mei, H. C.; Busscher, H. J. Artificial Channels in an Infectious Biofilm Created by Magnetic Nanoparticles Enhanced Bacterial Killing by Antibiotics. *Small* **2019**, *15* (39), e1902313.
(49) Chen, T.; Wang, R.; Xu, L. Q.; Neoh, K. G.; Kang, E.-T. Carboxymethyl Chitosan-Functionalized Magnetic Nanoparticles for Disruption of Biofilms of Staphylococcus Aureus and Escherichia Coli. *Ind. Eng. Chem. Res.* **2012**, *51* (40), 13164–13172.
(50) Quan, K.; Jiang, G.; Liu, J.; Zhang, Z.; Ren, Y.; Busscher, H. J.; van der Mei, H. C.; Peterson, B. W. Influence of Interaction between Surface-Modified Magnetic Nanoparticles with Infectious Biofilm Components in Artificial Channel Digging and Biofilm Eradication by Antibiotics in Vitro and in Vivo. *Nanoscale* **2021**, *13* (8), 4644–4653.
(51) Tong, F.; Wang, P.; Chen, Z.; Liu, Y.; Wang, L.; Guo, J.; Li, Z.; Cai, H.; Wei, J. Combined Ferromagnetic Nanoparticles for Effective Periodontal Biofilm Eradication in Rat Model. *Int. J. Nanomedicine* **2023**, *18*, 2371–2388.
(52) Vo, D.-T.; Sabrina, S.; Lee, C.-K. Silver Deposited Carboxymethyl Chitosan-Grafted Magnetic Nanoparticles as Dual Action Deliverable Antimicrobial Materials. *Mater. Sci. Eng. C Mater. Biol. Appl.* **2017**, *73*, 544–551.
(53) Ghaseminezhad, S. M.; Shojaosadati, S. A.; Meyer, R. L. Ag/Fe3O4 Nanocomposites Penetrate and Eradicate S. Aureus Biofilm in an in Vitro Chronic Wound Model. *Colloids Surf. B Biointerfaces* **2018**, *163*, 192–200.
(54) Mahmoudi, M.; Serpooshan, V. Silver-Coated Engineered Magnetic Nanoparticles Are Promising for the Success in the Fight against Antibacterial Resistance Threat. *ACS Nano* **2012**, *6* (3), 2656–2664.
(55) Nickel, R.; Kazemian, M. R.; Wroczynskyj, Y.; Liu, S.; van Lierop, J. Exploiting Shape-Selected Iron Oxide Nanoparticles for the Destruction of Robust Bacterial Biofilms - Active Transport of Biocides via Surface Charge and Magnetic Field Control. *Nanoscale* **2020**, *12* (7), 4328–4333.
(56) Li, J.; Nickel, R.; Wu, J.; Lin, F.; van Lierop, J.; Liu, S. A New Tool to Attack Biofilms: Driving Magnetic Iron-Oxide Nanoparticles to Disrupt the Matrix. *Nanoscale* **2019**, *11* (14), 6905–6915.
(57) Wang, X.; Wu, J.; Li, P.; Wang, L.; Zhou, J.; Zhang, G.; Li, X.; Hu, B.; Xing, X. Microenvironment-Responsive Magnetic Nanocomposites Based on Silver Nanoparticles/Gentamicin for Enhanced Biofilm Disruption by Magnetic Field. *ACS Appl. Mater. Interfaces* **2018**, *10* (41), 34905–34915.
(58) Ji, Y.; Han, Z.; Ding, H.; Xu, X.; Wang, D.; Zhu, Y.; An, F.; Tang, S.; Zhang, H.; Deng, J.; Zhou, Q. Enhanced Eradication of Bacterial/Fungi Biofilms by Glucose Oxidase-Modified Magnetic Nanoparticles as a Potential Treatment for Persistent Endodontic Infections. *ACS Appl. Mater. Interfaces* **2021**, *13* (15), 17289–17299.
(59) Li, L.-L.; Yu, P.; Wang, X.; Yu, S.-S.; Mathieu, J.; Yu, H.-Q.; Alvarez, P. J. J. Enhanced Biofilm Penetration for Microbial Control by Polyvalent Phages Conjugated with Magnetic Colloidal Nanoparticle Clusters (CNCs). *Environ. Sci. Nano* **2017**, *4* (9), 1817–1826.
(60) Zhang, C.; Du, C.; Liao, J.-Y.; Gu, Y.; Gong, Y.; Pei, J.; Gu, H.; Yin, D.; Gao, L.; Pan, Y. Synthesis of Magnetite Hybrid Nanocomplexes to Eliminate Bacteria and Enhance Biofilm Disruption. *Biomater. Sci.* **2019**, *7* (7), 2833–2840.

(61) Xu, Y.; Wang, K.; Zhu, Y.; Wang, J.; Ci, D.; Sang, M.; Fang, Q.; Deng, H.; Gong, X.; Leung, K. C.-F.; Xuan, S. Size-Dependent Magnetomechanically Enhanced Photothermal Antibacterial Effect of Fe3O4@Au/PDA Nanodurian. *Dalton Trans.* **2023**, *52* (46), 17148–17162.
(62) Bhuyan, T.; Simon, A. T.; Maity, S.; Singh, A. K.; Ghosh, S. S.; Bandyopadhyay, D. Magnetotactic T-Budbots to Kill-n-Clean Biofilms. *ACS Appl. Mater. Interfaces* **2020**, *12* (39), 43352–43364.
(63) Tran, H. Q.; Alam, H.; Goff, A.; Daeneke, T.; Bhave, M.; Yu, A. Multifunctional Fe3O4 Nanoparticles Filled Polydopamine Hollow Rods for Antibacterial Biofilm Treatment. *Molecules* **2023**, *28* (5). https://doi.org/10.3390/molecules28052325.
(64) Sun, B.; Sun, M.; Zhang, Z.; Jiang, Y.; Hao, B.; Wang, X.; Cao, Y.; Chan, T. K. F.; Zhang, L. Magnetic Hydrogel Micromachines with Active Release of Antibacterial Agent for Biofilm Eradication. *Adv. Intell. Syst.* **2024**, *6* (2). https://doi.org/10.1002/aisy.202300092.
(65) Mayorga-Martinez, C. C.; Zelenka, J.; Klima, K.; Kubanova, M.; Ruml, T.; Pumera, M. Multimodal-Driven Magnetic Microrobots with Enhanced Bactericidal Activity for Biofilm Eradication and Removal from Titanium Mesh. *Adv. Mater.* **2023**, *35* (23), e2300191.
(66) Dasgupta, D.; Peddi, S.; Saini, D. K.; Ghosh, A. Mobile Nanobots for Prevention of Root Canal Treatment Failure. *Adv. Healthc. Mater.* **2022**, *11* (14), e2200232.
(67) Hwang, G.; Paula, A. J.; Hunter, E. E.; Liu, Y.; Babeer, A.; Karabucak, B.; Stebe, K.; Kumar, V.; Steager, E.; Koo, H. Catalytic Antimicrobial Robots for Biofilm Eradication. *Sci. Robot.* **2019**, *4* (29), eaaw2388.
(68) Oh, M. J.; Babeer, A.; Liu, Y.; Ren, Z.; Wu, J.; Issadore, D. A.; Stebe, K. J.; Lee, D.; Steager, E.; Koo, H. Surface Topography-Adaptive Robotic Superstructures for Biofilm Removal and Pathogen Detection on Human Teeth. *ACS Nano* **2022**, *16* (8), 11998–12012.
(69) Sun, M.; Chan, K. F.; Zhang, Z.; Wang, L.; Wang, Q.; Yang, S.; Chan, S. M.; Chiu, P. W. Y.; Sung, J. J. Y.; Zhang, L. Magnetic Microswarm and Fluoroscopy-Guided Platform for Biofilm Eradication in Biliary Stents. *Adv. Mater.* **2022**, *34* (34), e2201888.
(70) Sun, B.; Guo, J.; Hao, B.; Cao, Y.; Chan, T. K. F.; Sun, M.; Sung, J. J. Y.; Zhang, L. Liquid-Bodied Antibiofilm Robot with Switchable Viscoelastic Response for Biofilm Eradication on Complex Surface Topographies. *Sci. Adv.* **2025**, *11* (11), eadt8213.
